# Molecular Signatures of HIV‐Induced Neuroinflammation and Neurodegeneration in Human Cerebral Organoids: Identification of Therapeutic Targets for NeuroHIV

**DOI:** 10.1002/advs.77954

**Published:** 2026-10-18

**Authors:** Karthick Chennakesavan, Malaroviyam Samikkannu, Shreyan Gupta, Olasunkanmi Israel Tayo, Arpan Acharya, James J. Cai, Siddappa N. Byrareddy, Thangavel Samikkannu

**Affiliations:** ^1^ Department of Pharmaceutical Sciences Irma Lerma Rangel College of Pharmacy Texas A&M University College Station Texas USA; ^2^ Department of Veterinary Integrative Biosciences School of Veterinary Medicine and Biomedical Sciences Texas A&M University College Station Texas USA; ^3^ Department of Pharmacology and Experimental Neuroscience College of Medicine University of Nebraska Medical Center Omaha Nebraska USA

**Keywords:** Combination Antiretroviral Therapy (cART), HIV‐associated neurocognitive disorders (HAND), Human cerebral organoids, Macaque brain, Neuroinflammation, Single nuclei multi‐omics, Synaptic dysfunction

## Abstract

HIV‐associated neurological complications remain a major concern in people living with HIV (PWH), even under effective viral suppression with combination antiretroviral therapy (cART), underscoring unresolved mechanisms driving HIV‐related neurodegeneration. To address this gap, we used HIV‐1‐infected human cerebral organoids (hCOs) containing microglia as a physiologically relevant three‐dimensional model of the central nervous system (CNS) and validated key findings in the brain region of simian immunodeficiency virus (SIV)‐infected, cART‐treated rhesus macaque model. Integrated single‐nucleus RNA sequencing with ATAC‐seq (multi‐omics), identifies cell‐type‐specific alterations in dual innate immune‐driven inflammatory signaling pathways across neural and glial populations in hCOs. Microglia were preferentially infected and activated, initiating IL‐17A–mediated cascades involving IFN‐γ/STAT1, CEBPB, and NF‐κB signaling. These pathways converged with cGAS‐STING and Notch–NEURL1 signaling, both associated with neuroinflammation and synaptic dysfunction. cART suppressed viral replication, reduced IL‐17A‐mediated neuroinflammation, improved mitochondrial function, and restored synaptic gene expression. Collectively, these findings identify dysregulation of IL‐17A, STAT1, CEBPB, and cGAS‐STING signaling as key molecular drivers of HIV‐associated neuroinflammation. They also establish a potential mechanistic link between IL‐17A signaling and microglial remodeling, providing a translational framework for the development of CNS‐targeted therapies in PWH.

## Graphical abstract

HIV preferentially infects microglia, activating IL‐17A‐driven IFN‐γ/STAT1, CEBPB, and NF‐κB signaling that converges with cGAS‐STING and Notch–NEURL1 pathways, promoting neuroinflammation, mitochondrial dysfunction, and synaptic impairment. cART suppresses viral replication, attenuates inflammatory signaling, and at least partially restores mitochondrial and synaptic function, highlighting potential therapeutic targets for HIV‐associated neurodegeneration.

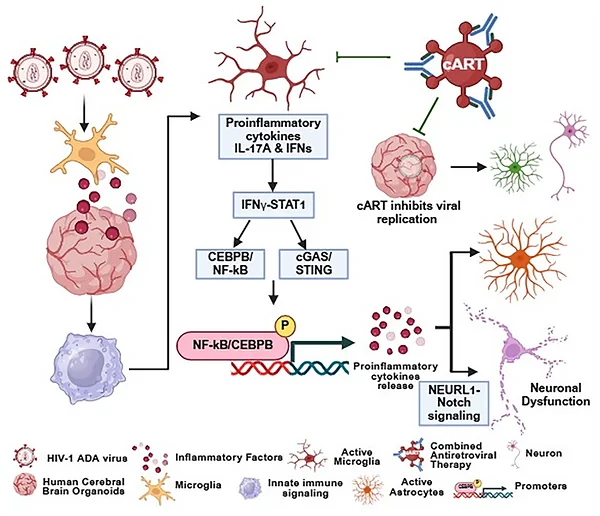

## 1 Introduction

According to the 2024 Joint United Nations Programme on HIV/AIDS (UNAIDS) report, an estimated 40.8 million people worldwide are living with HIV (PWH), with 1–2 million new infections occurring annually [1]. Despite the widespread availability of highly effective combination antiretroviral therapy (cART), only ∼60% of PWH achieve undetectable plasma HIV viral loads, mainly due to incomplete adherence, pharmacokinetic variability, drug resistance, or immune dysfunction [2]. HIV‐associated neurocognitive disorder (HAND) remains a major complication, affecting 30%–50% of PWH, most often in its milder forms, even in the long‐acting cART era. Persistent low‐level HIV replication in the brain, despite systemic viral suppression, is a recognized driver of chronic neuroinflammation [3].

Multi‐omics analysis has significantly advanced our ability to resolve cell‐type‐specific gene expression and molecular pathways, particularly when applied to human cerebral organoids (hCOs) [4, 5]. Integrating transcriptomic, immune response, and inflammatory profiles from single‐cell datasets enables a detailed dissection of cellular complexity in the human brain and the mechanisms driving neurodegenerative disease progression [5, 6]. hCOs recapitulate key features of the human central nervous system (CNS), encompassing diverse resident cell types such as neurons, astrocytes, oligodendrocytes, and microglia, while preserving spatial organization and higher‐order functional architecture [6, 7]. These characteristics make hCOs a powerful platform for modeling neurodevelopment, neurodegeneration, and intercellular interactions in both physiological and pathological contexts.

To elucidate HIV neuropathogenesis, it is essential to identify CNS‐resident cells that mediate immune responses. In the brain parenchyma, immune defense is primarily carried out by glial cells, which are long‐lived myeloid cells that reside permanently within brain tissue [8]. Microglia, the brain's resident macrophages, are highly susceptible to HIV infection and contribute to the establishment of long‐lived viral reservoirs. Once infected, microglia support persistent viral replication and initiate a chronic inflammatory milieu, releasing pro‐inflammatory cytokines and inducing type I interferon‐stimulated genes (ISGs) [8, 9]. These ISGs perpetuate inflammation, impair synaptic plasticity, and contribute to HAND and other neurodegenerative disorders [9]. In addition to microglia, CD4^+^ T cells play an important role in maintaining CNS immune surveillance and homeostasis. Brain‐resident CD4^+^ T cells resemble peripheral central memory T cells and remain functionally competent; however, their depletion during chronic simian immunodeficiency virus (SIV) infection is associated with heightened neuroinflammation. Restoration of CD4^+^ T memory stem cell homeostasis following antiretroviral therapy further underscores the importance of these cells in regulating CNS immune responses and viral pathogenesis [10, 11, 12].

One key regulator of neuroinflammation is CCAAT/enhancer‐binding protein‐β (CEBPB), also known as nuclear factor interleukin‐6 (NF‐IL6), a transcription factor central to inflammatory signaling in the CNS [13, 14]. CEBPB coordinates glial inflammatory responses and may interact with interleukin‐17A (IL‐17A), a pro‐inflammatory cytokine produced primarily by Th17 cells, to exacerbate neuroinflammation. IL‐17A synergizes with tumor necrosis factor (TNF) to increase production of chemokines such as CCL20, which recruit additional Th17 cells and promote HIV replication [13, 15, 16]. Notably, previous studies have shown that CEBPB regulates the expression of inflammation‐related factors, including monocyte chemotactic protein‐1 (MCP‐1), in HIV‐treated astrocytes and microglia [14, 17], and plays a dual role, propagating inflammation while also contributing to resolution during tissue repair [16, 18]. Specified persistent immune activation in HIV‐associated neuroinflammation despite cART, the CEBPB/IL‐17A axis represents a promising but underexplored therapeutic target.

Another candidate of interest is neuralized‐like protein 1A (NEURL1), an E3 ubiquitin‐protein ligase involved in Notch signaling and neuronal differentiation. In glial cells, NEURL1 plays a role in maintaining homeostasis and alters cellular CNS inflammatory responses [19]. Dysregulation of NEURL1, potentially via impaired Notch–JAG1 signaling, has been linked to glial vulnerability under pathological conditions such as HIV infection and tumor progression [20]. The Notch pathway also influences IL‐17A production [21], suggesting that the NEURL1–Notch–IL‐17A signaling pathway could represent a potential mechanism linking HIV infection to CNS inflammation and synaptic dysfunction. Despite its potential importance, NEURL1 remains poorly studied in the context of HIV neuropathogenesis.

In addition to viral and host factors, therapeutic regimens themselves may influence CNS inflammation. For example, the cART regimen ATRIPLA (Efavirenz, Emtricitabine, Tenofovir) can suppress NF‐κB activation, potentially reducing inflammatory signaling and preventing neuronal injury [3, 22]. However, dissecting these interactions in vivo remains challenging due to the complexity of CNS immune networks. Human cerebral organoids (hCOs), combined with advanced single‐nucleus multi‐omics approaches, provide a unique platform to investigate these pathways in a controlled yet physiologically relevant setting. In this study, we examine how cART‐suppressed HIV‐1 infection alters the expression and functional activity of NEURL1 and the CEBPB/IL‐17A signaling axis in hCOs and determine the downstream effects on neuroinflammation, mitochondrial function, and neuronal integrity. To validate these findings and assess cell‐type‐specific inflammatory transcriptomic changes, we integrated our organoid data with multi‐omic datasets generated from the brains of SIV‐infected, cART‐treated rhesus macaques. This complementary translational approach enables the identification of conserved molecular mechanisms underlying persistent neuroinflammation and highlights potential immunotherapeutic targets to mitigate HIV‐associated neuroinflammation and preserve cognitive function in PWH.

## 2 Results

### 2.1 Development, Maturation, and Differentiation of Human Cerebral Organoids (hCOs): Stem Cell Markers, Proliferation, and Glial‐Neuronal Characterization

The formation of hCOs is a complex process that begins with the derivation of cells from induced pluripotent stem cell lines (iPSCs). These cells self‐organize to form distinct brain regions within a 3D structure [23, 24]. In this study, human iPSCs (#iPS15, Alstem, USA) were differentiated into hCOs using previously established protocols [24]. Briefly, hiPSCs were first aggregated into embryoid bodies (EBs) with a uniform diameter of approximately 400–500 µm, exhibiting smooth and well‐defined spherical morphology. EBs were subsequently subjected to neural induction, followed by expansion through embedding in Matrigel and cultivation in expansion medium. During the induction and early expansion phases, EBs began to form neuroepithelial structures, which were evident as surface budding and progressive radial outgrowth beginning on days 7–10 (Figure 1A). These neuroepithelial regions increased in size and complexity, marking the transition toward organoid patterning. The maturation phase was initiated on day 11 by transferring organoids into maturation medium.

**FIGURE 1:**
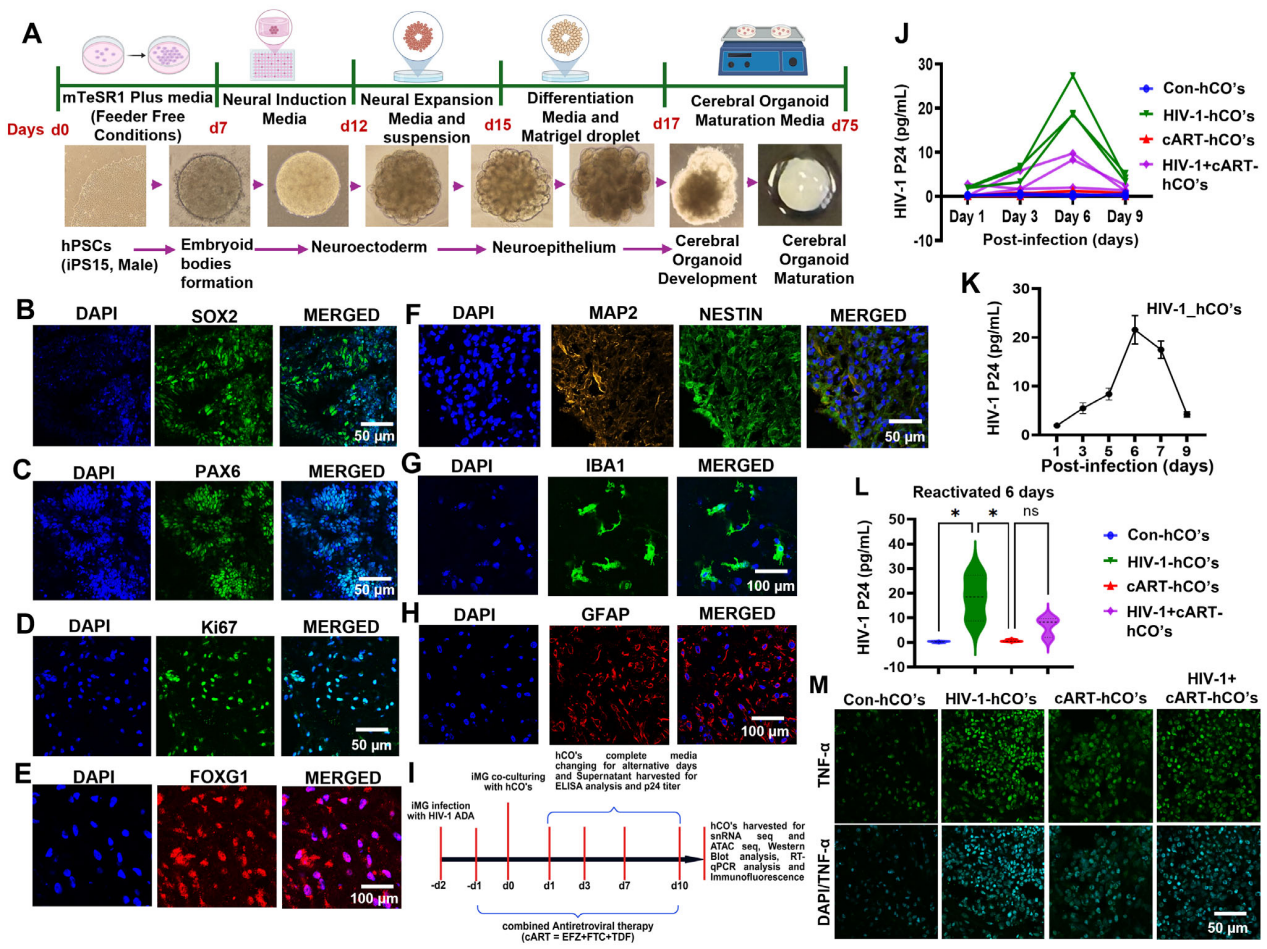
Generation and characterization of hCOs from iPS15 Line. (A) Diagrammatic representation illustrating the developmental stages of hCOs from iPS15 Lines. (B‐E) Immunostaining analysis of hCOs at 60 days to assess the expression of stem cell proliferation markers, SOX2, PAX6, Ki67, and FOXG1, respectively. (F–H) Characterization of glial and neuronal cell populations within 60‐day‐old hCOs using specific markers: (F) NESTIN and MAP2, a neuronal marker representing mature neurons and dendritic structures; (G) Iba1, a microglial marker; (H) GFAP, an astrocytic marker highlighting glial cell maturation. Scale bars, 50 and 100 µm. (I) schematic representation illustrating the experimental setup in which hCOs are infected with the HIV‐1‐ADA virus strain. (J‐L) HIV p24 ELISA analysis was performed on supernatant samples collected from different experimental groups, including control, HIV‐1 infected, and with or without cART treatment. A total of 6–8 individual hCO's were pooled for each experimental group. All experiments were conducted using organoids generated from at least three independent experiments. (M) Immunofluorescence of hCOs stained with anti‐TNF‐α antibodies (green), a pro‐inflammatory marker, and with DAPI, overexpression of TNF‐α was observed in HIV‐1‐infected organoids compared to controls. Data are represented as mean ± SD (*n* = 3), three independent experiments. One‐way ANOVA followed by Tukey's multiple comparison test. Statistically significant at ^*^ *p* < 0.01, and ns‐Not significant. iMG‐ HIV‐ infected microglia, EFZ ‐ Efavirenz, FTC ‐ Emtricitabine, TDF ‐ Tenofovir disoproxil fumarate.

By day 60, hCOs displayed morphological features consistent with maturation, including dense cellular cores and translucent peripheral regions. On day 60, hCOs showed robust differentiation into multiple cell types, with a consistent diameter of 2.0 to 2.5 mm. Immunofluorescence analysis confirmed the presence of neuronal, astrocytic, and microglial populations, as indicated by positive staining for MAP2 (mature neurons) and Nestin (neural progenitors/immature neurons), GFAP (astrocytes), and Iba1 (microglia) (Figure 1). The development and maturation of brain organoids over 60 days were monitored using stem cell and proliferation markers (Figure 1A–H). In parallel, we evaluated whether the established brain organoids accurately replicate key aspects of human brain development. To achieve this, we performed immunofluorescence staining for stem cell proliferation markers, including SOX2, PAX6, Ki67, FOXG1, and Nestin, which were observed on day 60. SOX2, PAX6, and Ki67 expression were developed in neural progenitor cells (NPCs) within the proliferative zones of the cerebral organoids, marking active neurogenesis and supporting the expansion and migration of neuronal precursors. At this stage, the organoids displayed well‐defined neuroepithelial architecture, with expanding NPC populations driving early cortical‐like patterning, as shown in Figure 1B–D.

We next characterized neuronal and glial differentiation in hCOs by assessing lineage‐specific markers. FOXG1 expression was detected in day 60 organoids, indicating neuronal maturation (Figure 1E). Neuronal differentiation was further assessed using Nestin and MAP2; Nestin expression marked neural progenitors, whereas MAP2 expression increased with maturation. As differentiation progressed, the forebrain marker FOXG1 and the mature neuronal marker MAP2 showed increased expression in cerebral organoids at days 30 and 60 compared to earlier developmental stages, reflecting enhanced neuronal differentiation and dendritic maturation during prolonged organoid development (Figure S1A,B). MAP2 neurons display elongated, branched processes indicative of functional neuronal maturation (Figure 1F). The microglial marker Iba1 was expressed in the matured organoids, with Iba1^+^ cells predominantly localized to outer regions and displaying ramified, homeostatic morphologies (Figure 1G). Astrocyte differentiation was confirmed by GFAP expression, with GFAP^+^ cells distributed in the organoids and exhibiting characteristic stellate morphology, consistent with the establishment of a supportive glial network (Figure 1H).

### 2.2 Incorporation of HIV‐1 ADA Virus‐Infected Microglia Into hCOs

Our primary objective in developing the hCOs model is to investigate HIV‐associated neuroinflammation in a system that closely mimics in vivo HIV pathogenesis. A key element of HIV‐1 neuropathogenesis is the presence of HIV‐infected human microglia derived from induced pluripotent stem cells (iPSCs) [25, 26]. HIV‐1 exhibits extensive genetic variability, leading to significant differences among viral strains in their biological properties, including replicative capacity, syncytium‐inducing potential, and tropism for specific target cells. Most primary isolates can infect both cell types; however, individual strains often display a distinct preference for either lymphocytes or macrophages, reflecting their coreceptor usage and influencing clinical progression and neuropathogenesis [27]. Among these, the HIV‐1 ADA strain is a well‐characterized, macrophage‐tropic and neurovirulent subtype B isolate, frequently used as a model to study HIV‐1 infection in the CNS and to evaluate antiretroviral drug efficacy in macrophage‐based systems [28, 29].

To model this process under physiologically relevant conditions, we generated human microglia from induced pluripotent stem cells (iPSCs) and infected them with HIV‐1 ADA strain. To replicate in vivo HIV neuropathogenesis, infected microglia were surface‐seeded with hCOs, and the cultures were maintained for 9 days postinfection (dpi), following treatment with cART (Figure 1I). HIV‐1 infection was confirmed through HIV p24 ELISA analysis (Figure 1J–L), compared with control hCOs. As shown in Figure 1J, HIV p24 levels were significantly elevated in the infected hCOs on dpi 6 compared to earlier time points. The gradual increase in p24 protein levels over time reflects the progressive establishment and amplification of HIV‐1 replication within the cerebral organoids, confirming successful and sustained viral infection. To evaluate the efficacy of cART, supernatants were collected every 3 days and analyzed for p24 levels by ELISA. cART treatment significantly reduced p24 antigen levels compared with HIV‐1‐infected controls (Figure 1K,L). Immunostaining further confirmed that cART therapy markedly decreased TNF‐α expression in HIV‐1 infected hCOs relative to other groups (Figure 1M).

### 2.3 Single‐Cell Transcriptional and Chromatin Accessibility Changes in HIV‐1 ADA‐infected hCOs Using Integrated Multi‐Omics Analysis

To evaluate multiplexed, long‐term organoid modeling under HIV‐1 infection and cART, samples were pooled across all conditions before single‐cell encapsulation (Figure 2A and Methods). We performed batch correction on both snATAC‐seq and snRNA‐seq datasets using the R package Harmony Integration. Subsequently, Seurat v5 and Signac v1.14 R packages were employed to characterize cellular composition in the snRNA‐seq data, annotate cells based on transcriptional profiles, and guide the snATAC‐seq analysis (Figure 2B,C). The snRNA‐seq analysis identified all major cell types within the cerebral organoids, using lineage‐specific markers (Figure 2B). Detected cell populations included neural stem cells (NSCs), midbrain dopaminergic neurons (mDNs), neurons, cycling radial glial cells (cRGCs), oligodendrocytes (oligos), astrocytes (astro), microglia, and oligodendrocyte precursor cells (OPCs). These populations were visualized using Uniform Manifold Approximation and Projection (UMAP; Figure 2B,C).

**FIGURE 2:**
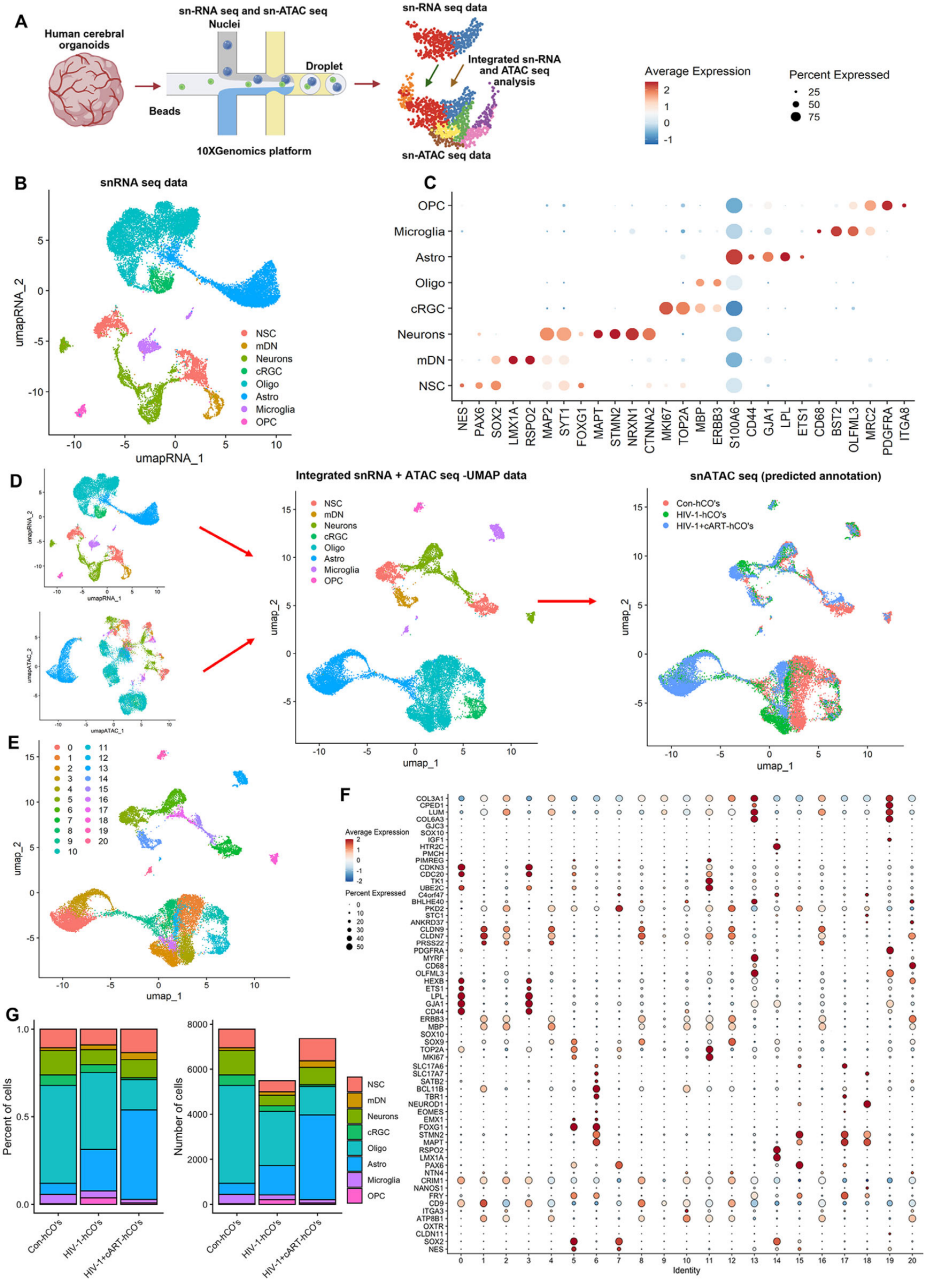
Single‐cell transcriptional and chromatin accessibility profiling on HIV‐1 infected hCOs with cART. (A) Graphical illustration of experimental methodology. Single‐nuclei RNA sequencing (snRNA‐seq) and ATAC‐seq were performed on ∼60‐day‐old hCOs following HIV‐1 infection with cART regimen (*n* = 8–12 brain organoids/group). (B) UMAP plots of snRNA‐seq dataset. NSC‐ neural stem cells, mDN – midbrain dopaminergic neurons, Neurons, cRGC‐ cycling radial glial cells, oligo – oligodendrocytes, astro – astrocytes, microglia, OPC‐ oligodendrocyte progenitor cells. (C) Dot plot of snRNA‐seq dataset showing gene expression patterns of cluster‐enriched markers. The diameter of the dot corresponds to the proportion of cells expressing the indicated gene, and the density of the dot corresponds to the average expression across all cell types. (D) Multi‐omics integration strategy for processing the snATAC‐seq dataset. Following integrated snRNA‐seq and snATAC‐seq were filtered using a 97% prediction score threshold for cell‐type assignment. (E) UMAP plot of snATAC‐seq dataset with gene activities‐based cell type markers. (F) The diameter of the dot corresponds to the proportion of cells with detected activity of the indicated gene, and the density of the dot corresponds to the average gene activity relative to all cell types. (G) Proportion of cell numbers and cell percentages shown in c, in different treatment conditions.

snATAC‐seq captures the chromatin‐accessibility profile of individual cells [30]. Since cell‐type‐specific chromatin accessibility remains understudied, we leveraged our annotated snRNA‐seq dataset to predict cell identities in the snATAC‐seq data using Seurat's label‐transfer framework. We generated a gene‐activity matrix representing chromatin accessibility across gene bodies and promoters from the snATAC‐seq data as the query, then identified transfer anchors between the snRNA‐seq reference and this matrix. The resulting prediction scores indicated that most snATAC‐seq cells were confidently matched to a single cell type (Figure 2D). Comparison of these label‐transfer predictions with curated annotations of unsupervised clusters (Figure 2E,F) confirmed representation of all major cell types. It demonstrated that snATAC‐seq provides resolution comparable to snRNA‐seq for cell‐type assignment. Interestingly, snATAC‐seq identified 20 subpopulations within the cerebral organoid clusters (Figure 2E), and the proportions of these clusters remained consistent across different treatment conditions (Figure 2F).

Following HIV‐1 infection, we observed notable shifts in cell‐type composition within the cerebral organoids. Microglia, astrocytes, oligodendrocytes, neural stem cells (NSCs), oligodendrocyte precursor cells (OPCs), mature dopaminergic neurons (mDNs), cortical radial glial cells (cRGCs), and neurons accounted for 3.89%, 23.69%, 43.83%, 9.01%, 3.75%, 2.71%, 4.51%, and 8.57% of the total cell population of HIV‐1 infected hCOs, respectively (Figure 2G and File S1). Compared with uninfected controls, HIV‐1 infection resulted in a noticeable increase in OPCs and mDNs, suggesting that viral exposure alters neural lineage dynamics and cellular differentiation trajectories. The expansion of OPCs may reflect a compensatory response to HIV‐induced cellular stress or myelin‐related dysfunction, whereas the increased proportion of mDNs may indicate selective vulnerability, altered maturation, or survival of specific neuronal subpopulations.

### 2.4 Dual Innate Immune Pathways Drive Robust HIV‐1–Associated Inflammatory Responses in Human Cerebral Organoids

To assess the molecular impact of HIV‐1 infection in hCOs, we performed differential gene expression (DEG) analysis using fgsea (R) [31, 32] across annotated cell types, comparing HIV‐1‐infected samples to uninfected controls and HIV‐1 + cART‐treated groups. This revealed widespread transcriptional changes across neurons and glial cells (Data File S1). Remarkably, genes involved in immune activation and cellular stress were significantly upregulated, highlighting the persistent inflammatory state associated with HIV‐1‐neuropathogenesis. Enrichment analysis of differentially expressed genes revealed significant enrichment of key biological pathways, including cytokine and interferon signaling, Toll‐like receptor (TLR) signaling, mitochondrial electron transport, and glial cell development and differentiation. These pathway alterations were prominent in astrocyte and microglial populations (Figure 3A–D) and were consistently observed across all cell subtypes (Figure S2A–H). Collectively, these pathways align with the chronic immune activation and metabolic stress characteristic of HIV‐1‐infected hCOs. Mitochondrial dysfunction, reflected by changes in genes involved in electron transport, may underlie cellular energy deficits and oxidative stress. Furthermore, dysregulation of glial cell development pathways indicates potential disturbances in astrocyte and oligodendrocyte function, which may contribute to impaired neuro‐glial homeostasis. The top 10 differentially expressed genes (ranked by adjusted p‐value and fold change) in each major cell type are presented in Figure S3A, providing a comprehensive view of the transcriptional alterations underlying these functional shifts.

**FIGURE 3:**
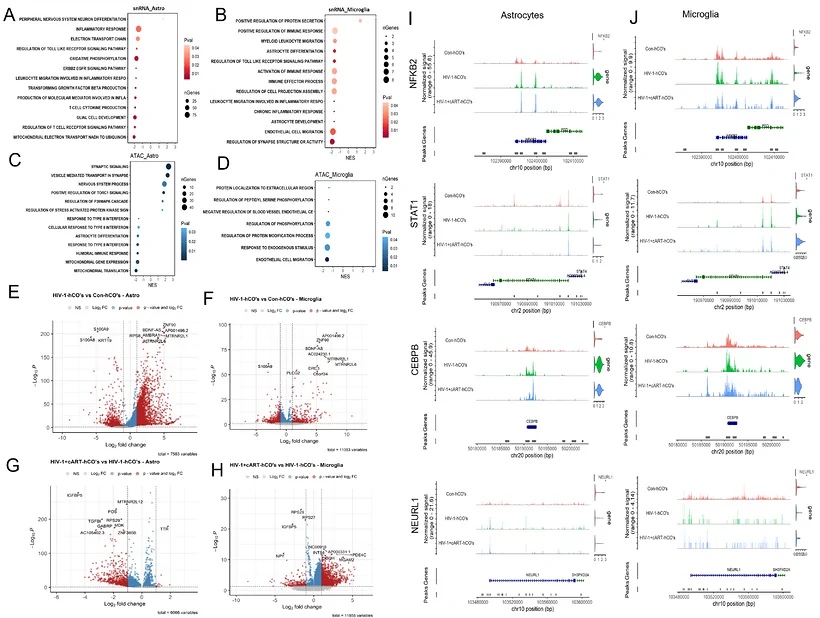
Single‐nucleus RNA‐seq and ATAC‐seq reveal differential chromatin accessibility and transcription factor regulation in HIV‐infected brain organoids. Functional enrichment analysis of differentially expressed genes. (A,B) Top 5 GO terms for the DEGs based on snRNA‐seq (BPs). (C,D) Top 5 GO terms for DEGS based on ATAC scores from different cell type including astrocytes and microglia of HIV‐1 vs. Control. GO, Gene Ontology; BP, biological process; adjusted p‐value. (E‐H) Volcano plot of genes that are significant (*p * ≤ 0.05; Wilcoxon rank sum test, Bonferroni‐adjusted) different when comparing HIV‐1 infected vs. Control (E‐F) and HIV‐1 + cART vs. HIV‐1 infection (G‐H). X‐axis displays the log2 fold‐change of each significant gene. (I‐J) Single‐nucleus ATAC‐seq was performed on cerebral organoids under control, HIV‐1 infected, and cART‐treated conditions to assess chromatin accessibility at the single‐cell level. Peak intensities represent open chromatin regions, with higher peaks indicating greater accessibility. Analysis was performed across major cell types, including microglia and astrocytes. Differential peak analysis revealed significant changes in regulatory elements, particularly at loci associated with immune‐related transcription factors, including NFKB2 (targeted gene locus on Chr 10), NEURL1 locus on Chr 10, CEBPB locus on Chr 20, and STAT1 locus on Chr 2.

Similarly, we performed single‐nuclei ATAC‐seq (snATAC‐seq), and the data showed differential chromatin accessibility across conditions and cell types (Figure S3B) using fgsea in R and the Molecular Signatures Database (MSigDB) v2024.1. In microglia, top‐enriched pathways included TLR signaling and TGF‐β mediated responses and regulatory pathways, whereas astrocytes exhibited altered chromatin accessibility at genes associated with synaptic signaling. Volcano plot analysis revealed that the DAMP‐related genes S100A8 and S100A9 were significantly downregulated in astrocyte and microglial populations of HIV‐1 infected hCOs. Notably, their expression levels were restored to near‐control levels following cART treatment (Figure 3E–H). Expression patterns in the remaining cell types are presented in Figure S4. In addition to several nuclear genes, MTRNR2L1, MTRNR2L6, AMBRA1, and ZNF90 were upregulated in HIV‐1‐infected hCOs. The altered expression of MTRNR2L1 and MTRNR2L6, genes likely reflecting a mitochondrial stress‐adaptive response, results in disrupted oxidative phosphorylation. Treatment with cART significantly reduced this expression, particularly in astrocytes, oligodendrocytes, and cRGC (Figure 3E–H and Figure S4).

To further elucidate transcriptomic inflammatory profiles changes, we performed differential peak accessibility analysis to identifying prominent transcriptional regulators such as NF‐κB2, NEURL1, CEBPB, and STAT1 showing significant differential accessibility peaks in astrocytes and glial cells (Figure 3I,J), neurons and oligos (Figure S5). Additional analysis revealed that altered accessibility in immune‐associated transcription factors, including STAT3, STAT4, IRF3, IRF9, and TFEB (Figure S6), was increased among all cell types. Moreover, we observed a significant reduction in S100A8 and S100A9 gene expression and chromatin accessibility in HIV‐1 infected hCOs compared to other experimental groups, suggesting that these changes may exert anti‐HIV effects in microglia, astrocytes, and oligodendrocytes (Figure S6). Together, our transcriptomic data reveal widespread, cell‐type‐specific immune activation and altered inflammatory response to HIV‐1 which was inhibited by cART treatment. Recent studies using primary DCs provide evidence that S100A9 suppresses HIV replication by inhibiting reverse transcription [33]. Consistent with this, mature monocyte‐derived Langerhans cells exhibit reduced intracellular S100A9 levels and increased susceptibility to HIV‐1 infection [34], underscoring that HIV‐1 infection outcomes can be highly cell‐type‐dependent. However, the individual roles of S100A8/A9 proteins in regulating HIV‐1 replication in primary cells remain poorly understood.

KEGG analysis revealed significant dysregulation in multiple signaling cascades, including chemokine, TGF‐β, Type I interferon, and oxidative phosphorylation pathways, when comparing HIV‐1‐infected hCOs to both control and cART‐treated groups (Figure 4A–D). Additionally, complementary GO enrichment analysis further indicated that key regulators of T‐cell activation and inflammatory responses are selectively upregulated in infected hCOs (Data File S1). Notably, IL‐17A, CEBPB, STAT1, FOXO3, and CSF1R are enriched in cytokine‐cytokine receptor interaction (Figure S7A). Notably, IGF2R, previously linked to microglial activation and HIV‐1 persistence in the CNS, was upregulated, while SLC11A1, a HAND‐associated marker implicated in synaptic dysfunction and apoptosis, emerged as a key feature in our integrated multi‐omics analysis (Data File S1). Remarkably, many DEGs in infected‐hCOs mapped to antiviral defense, innate immune regulation, and interferon‐mediated pathways, with a pronounced signature of proinflammatory cytokine and interferon‐γ signaling observed in the current study (Figure 4E,F, and Figure S7B).

**FIGURE 4:**
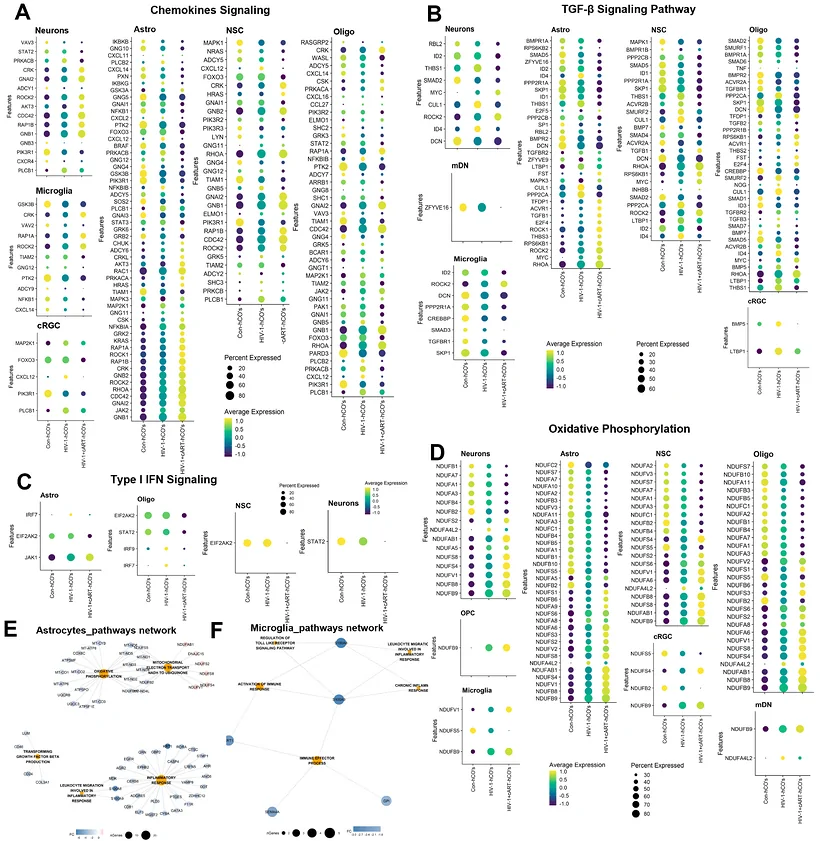
Functional enrichment analysis of upregulated genes in HIV‐1‐infected hCOs, highlighting key biological processes and pathways. (A–D) Kyoto Encyclopedia of Genes and Genomes (KEGG) pathways, offering a comprehensive view of the molecular mechanisms altered by HIV‐1‐infection. This analysis aids in identifying transcriptional regulators, cellular responses, and metabolic pathways affected by viral infection. (A) chemokines signaling, (B) TGF‐β signaling pathways, (C) oxidative phosphorylation, and (D) Type I IFN signaling. (E‐F). Bidirectional signaling pathways between astrocytes and microglia regulate neuroinflammatory outcomes.

### 2.5 Impact of HIV‐1 Infection and cART Treatment on Neuroinflammatory Signaling in Human Cerebral Organoids

Single‐nucleus RNA sequencing (snRNA‐seq) revealed that HIV‐1‐infected hCOs exhibit a pronounced inflammatory and immune activation signature compared with controls. HIV‐1 infection increased the expression of key transcriptional regulators, including ATF4, CEBPB, and CREB5, along with interferon‐stimulated genes such as IFI6 and IFITM1 (Figure 5A). In addition, markers associated with immune recruitment and inflammatory signaling, including CCL2, STAT1, and STAT3, were significantly upregulated, indicating a robust pro‐inflammatory response. Upregulation of NFKB1 and NFKB2 suggested activation of the NF‐κB pathway, while increased expression of multiple SMAD family members (SMAD3, SMAD4, SMAD6, SMAD7, SMAD9) implicated TGF‐β‐associated signaling in the neuroinflammatory process (Figure 5B). The overlap in cell‐type‐specific transcriptional changes, particularly in pathways associated with inflammation, immune activation, and neuronal dysfunction, underscores a conserved cellular response to HIV‐1 infection and cART treatment.

**FIGURE 5:**
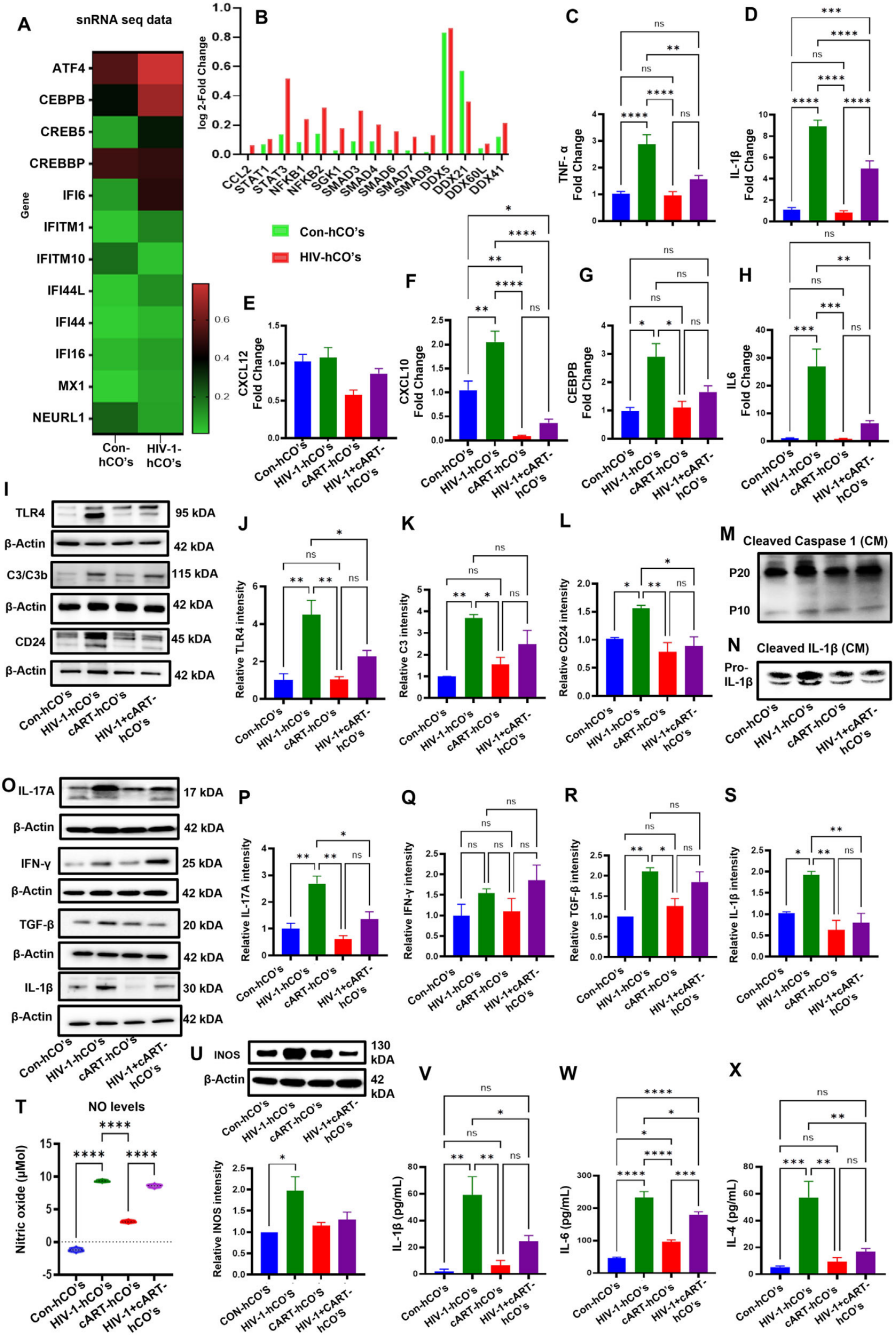
Exploration of HIV‐1 infection progression in hCOs with and without cART treatment. (A) Heat map showing the expression of proinflammatory marker genes in HIV‐1‐infected hCOs vs Control hCOs using snRNA sequencing analysis. The graph represents log2FC ratios of [HIV‐1‐hCO/control]. Log‐transformed, normalized expression values were averaged across samples per group and visualized using the heatmap R package. Row‐wise scaling was applied, and a diverging color scheme (blue to red) highlights differential expression. (B) RNA‐seq analysis showing the expression levels of CCL2, STAT1, STAT3, NFKB1, NFKB2, SGK1, SMAD, and DDX genes in HIV‐1‐infected hCOs vs control hCOs. (C‐H) mRNA expression of pro‐inflammatory cytokines, TNF‐α, IL‐1β, IL‐6, CEBPB, CXCL10, CXCL12 were analyzed using Real time qPCR analysis in HIV‐1 infected hCOs with or without cART. Fold changes were calculated using the formula 2‐ΔΔCT method. (I‐L) Immunoblot analysis was performed to assess the expression of key inflammatory and immune response proteins in HIV‐1‐infected hCOs with or without cART treatment. (J) TLR4, (K) C3/C3b, (L) CD24. (M) Cleaved caspase 1, (N) Cleaved IL‐1β were measured from conditioned media of different groups. (O) Western blot, left panel, and the levels of (P) IL‐17A, (Q) IFN‐γ, (R) TGF‐β, (S) IL‐1β, (U) iNOS, were quantified from 3 independent blots with respective bar graphs (right panels). Band intensities were measured and normalized to that of the respective β‐actin. (T) Nitrite levels were quantified in supernatant media collected at day 6 postinfection from different experimental groups. (V–X) Released level of IL‐1β, IL‐6 and IL‐4 was further quantified using ELISA analysis. Data are represented as mean ± SD (*n* = 3), three independent experiments. One‐way ANOVA followed by Tukey's multiple comparison test. ^*^ *p* < 0.05, ^**^ *p* < 0.01, ^***^ *p* < 0.001, ^****^ *p* < 0.0001 are represented as statistically significant, ns, Not significant.

Consistent with the transcriptomic analyses, qPCR validation confirmed significant upregulation of proinflammatory cytokines and immune‐response genes, including TNF‐α, IL‐1β, IL‐6, CEBPB, and C‐X‐C motif chemokine ligand 10 (CXCL10), in HIV‐1‐infected hCOs compared with uninfected controls (Figure 5C–H). Collectively, these findings support the presence of a robust inflammatory milieu within infected organoids and demonstrate that HIV‐1 infection promotes sustained activation of neuroimmune pathways that may contribute to neuronal dysfunction and the pathogenesis of NeuroHIV.

The inflammatory markers evaluated in this study represent endpoint measurements collected on day 9 following HIV‐1 infection and subsequent cART exposure, providing a snapshot of the cumulative molecular and cellular alterations that occur during the early stages of viral infection and therapeutic intervention. These findings indicate that HIV‐1 induces persistent alterations in cellular signaling networks that remain detectable despite antiretroviral treatment. Notably, several dysregulated genes and pathways were shared across multiple neural and glial cell populations, suggesting coordinated neuroimmune responses within the organoid microenvironment. Although the endpoint measurements do not capture the temporal dynamics of inflammatory signaling, they reflect the integrated outcome of HIV‐1‐induced neuroimmune activation and the extent to which cART modulates these responses at this defined stage.

### 2.6 HIV‐1 Promotes TLR4‐ and IL‐17A‐Mediated Neuroinflammatory Responses in Human Cerebral Organoids

Emerging studies have shown that Toll‐like receptor (TLR) signaling in the brain initiates an innate immune response primarily through microglia and astrocytes, contributing to neuroinflammation [35]. Among TLRs, TLR4 is distinct in activating both MyD88‐ and TRIF‐dependent pathways, resulting in amplified and prolonged inflammatory signaling. TLR4 is also more broadly expressed in CNS cell types and has been strongly associated with HIV‐induced neuroinflammation [36], making it a significant focus of this study. Here, we investigate how TLR4 activation in microglia drives pro‐inflammatory cascades and examine the role of complement pathways and immune mediators in regulating these responses. Western blot analysis revealed significantly elevated levels of TLR4, C3/C3b, and CD24 in HIV‐1‐infected human cerebral organoids (hCOs), indicative of a sustained neuroinflammatory response (Figure 5I–L). Similarly, immunostaining data showed increased CD68 expression in HIV‐1‐infected hCOs (Figure S8), indicating dysregulated neuroimmune signaling and heightened microglial activation. Interestingly, 9‐day cART treatment significantly reduced proinflammatory cytokine expression, TLR4 signaling, and CD24, an immune checkpoint molecule, in HIV‐1‐infected hCOs, while no significant changes were observed in the C3 complement system (Figure 5I–L). These findings suggest that cART not only suppresses HIV‐1 replication but also attenuates the inflammatory response associated with HIV‐1 infection. The reduction in proinflammatory cytokine levels suggests that cART may help restore immune homeostasis and mitigate the neuroinflammatory effects seen in HAND [37, 38].

Furthermore, immunoblot revealed that HIV‐1 infection robustly triggered neuroinflammatory responses in hCOs, as evidenced by significantly elevated levels of IL‐17A, transforming growth factor‐β (TGF‐β), and interferon‐γ (IFN‐γ) (Figure 5O–S). To further characterize the inflammatory milieu, we assessed levels of cleaved caspase‐1 and cleaved interleukin‐1β (IL‐1β) in the supernatant collected from HIV‐1‐infected organoids with cART treatment on day 6 post‐infection (dpi); both cleaved forms of caspase (P10 and P20) and cleaved IL‐1β were significantly increased in HIV‐1‐infected hCOs compared to controls (Figure 5M,N). Additionally, we assessed nitrite levels and inducible nitric oxide synthase (iNOS) expression as markers of reactive nitrogen species (RNS) production. HIV‐1‐infected hCOs exhibited significantly elevated nitrite levels (Figure 5T), while immunoblot analysis demonstrated a corresponding increase in iNOS expression (Figure 5U), indicating enhanced nitric oxide production and activation of oxidative and nitrosative stress pathways. The concomitant elevation of nitrite and iNOS suggests persistent inflammatory signaling that drives excessive RNS generation, a mechanism known to promote mitochondrial dysfunction, cellular injury, and neurotoxicity. These findings further support the existence of a heightened oxidative and nitrosative stress environment in HIV‐1‐infected organoids, which may contribute to neuronal damage and exacerbate neuroinflammatory processes.

In parallel, immunofluorescence analysis revealed increased co‐localization of microglia with IL‐17A expression in HIV‐1‐infected brain organoids compared with uninfected controls, indicating enhanced microglia‐associated IL‐17A signaling in response to HIV‐1 infection (Figure S9). This observation is consistent with the activation of proinflammatory microglial phenotypes and suggests a role for IL‐17A‐mediated neuroimmune signaling in sustaining chronic inflammation within the infected organoid microenvironment. Notably, cART treatment significantly reduced IL‐17A expression (Figure 5P), highlighting its potential to mitigate HIV‐1‐induced neuroinflammation and oxidative stress, thereby limiting neuronal injury and reducing the risk of neurocognitive impairment [38].

ELISA analysis further reveals that protein levels of IL‐1β, IL‐6, and IL‐4 were correspondingly increased during HIV‐1 infection (Figure 5V–X). These analyses were shown in supernatant collected from HIV‐1‐infected organoids with cART treatment on day 6 post‐infection. Consistent with our qPCR and Western blot findings, both assays confirmed the overexpression of these cytokines at the gene and protein levels. Notably, cART treatment helped restore homeostatic balance by reducing HIV‐associated neuroinflammation and microglial activation, as demonstrated in this study.

### 2.7 IL‐17A Promotes Neuroinflammation Through Modulation of CSF1R and STAT1 Signaling in hCOs

IL‐17A stimulates macrophages to secrete granulocyte colony‐stimulating factor (G‐CSF), monocyte chemoattractant protein‐1 (MCP‐1), and CXC chemokines, thereby promoting the recruitment and activation of monocytes and neutrophils at sites of inflammation [39]. In line with this mechanism, we assessed the expression of CSF1R (colony‐stimulating factor 1 receptor) and STAT1 (signal transducer and activator of transcription 1), both of which play central roles in inflammatory signaling and immune responses. We observed overexpression of phospho‐STAT1(Y701) and CSF1R in HIV‐1‐infected hCOs compared to controls (Figure 6A,B–D), suggesting that HIV‐1 infection triggers IL‐17A‐mediated signaling through CSF1R and STAT1.

**FIGURE 6:**
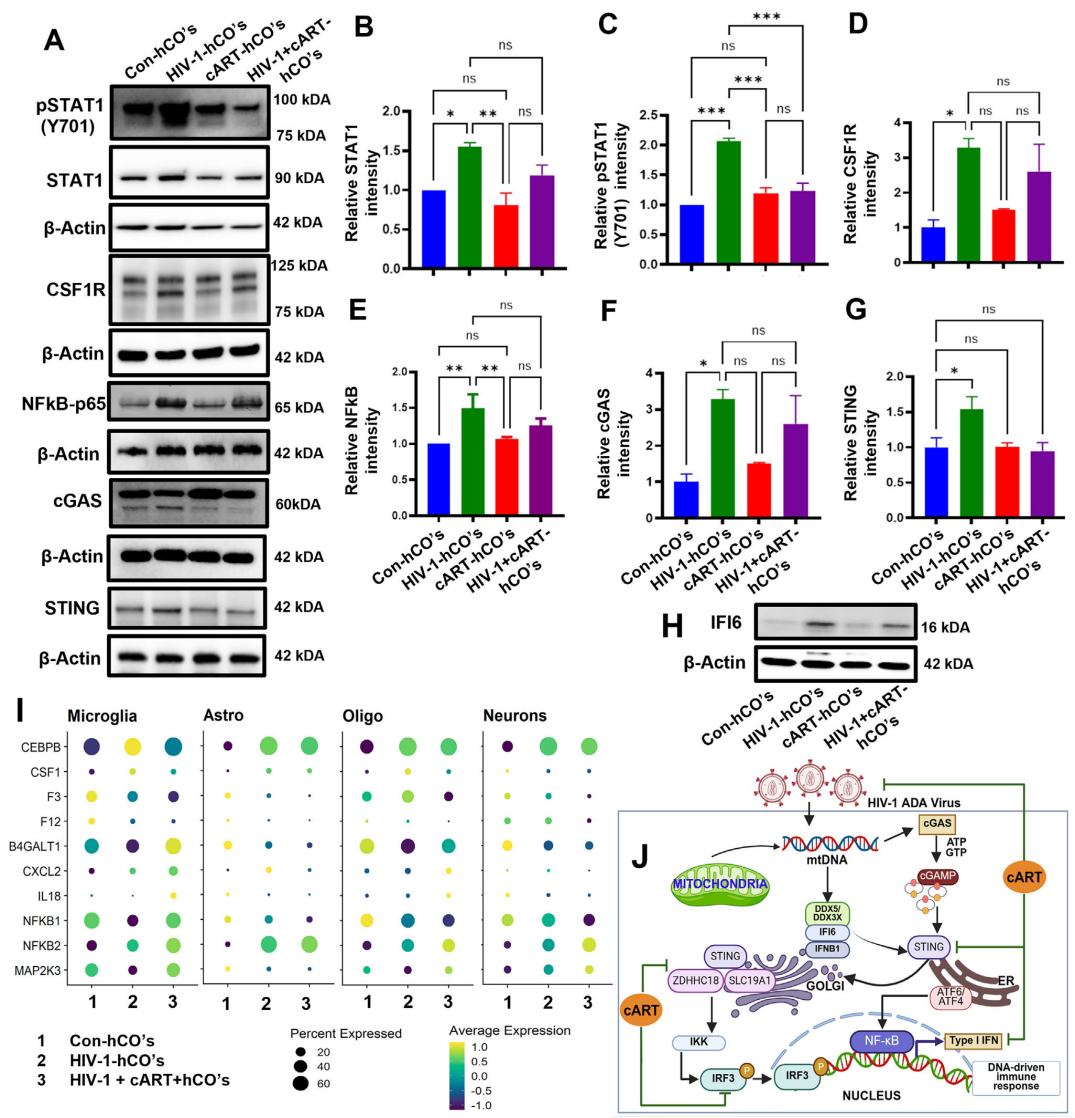
Impact of cART on STAT1 and cGAS/STING Signaling in HIV‐1‐infected brain organoids. (A‐G) Western blot analysis (left panels) of key immune signaling molecules, including (B) STAT1, (C) p‐STAT1 (Y701), (D) CSF1R, (E) NFKB‐P65, (F) cGAS, and (G) STING, in HIV‐1‐infected organoids with or without cART treatment, the quantified band intensities are displayed in the right panels. (H) Western blot analysis of IFI6 expression in HIV‐1‐infected hCOs compared to control and cART treatment. β‐Actin served as an internal control. (I) Integrated snRNA‐seq and snATAC‐seq analysis identified expression profiles of selected inflammatory markers across the various cell types among different groups. (J) Schematic illustration depicting HIV‐1‐induced activation of the DNA‐sensing innate immune pathway. HIV‐1 induced mtDNA activates cGAS‐STING‐dependent innate immune signaling. HIV‐1 ADA infection triggers mitochondrial DNA release, activating the cGAS‐STING pathway and downstream IRF3 and NF‐κB signaling, leading to type I interferon production. cART modulates STING signaling and attenuates DNA‐driven immune responses. Data are represented as mean ± SD (*n* = 3), three independent experiments. One‐way ANOVA followed by Tukey's multiple comparison test. ^*^ *p* < 0.05, ^**^ *p* < 0.01, ^***^ *p* < 0.001, ^****^ *p* < 0.0001 are represented as statistically significant, ns, Not significant.

When HIV‐1 infected hCOs were treated with cART, normalization of phospho‐STAT1 expression was observed, suggesting that antiretroviral therapy can effectively reduce IL‐17A levels. The normalization of these signaling pathways in the presence of cART underscores the therapeutic potential of antiretroviral drugs to reverse IL‐17A–mediated neuroinflammation in HAND and to attenuate downstream NF‐κB signaling via STAT1 activation (Figure 6A,E).

Moreover, HIV‐1 infection induced a marked upregulation of cyclic GMP‐AMP synthase (cGAS) and stimulator of interferon genes (STING) in hCOs, indicating activation of the cytosolic DNA‐sensing pathway. This finding suggests that HIV‐1 infection triggers innate immune surveillance mechanisms that detect viral nucleic acids and initiate downstream inflammatory signaling cascades. Activation of the cGAS–STING pathway is known to promote the production of type I interferons and proinflammatory cytokines during HIV‐1 infection, potentially exacerbating neuroimmune dysregulation and tissue injury within the CNS [40, 41, 42]. Elevated expression of cGAS and STING in HIV‐1‐infected hCOs further suggests that persistent sensing of viral DNA or damaged mitochondrial DNA may sustain a proinflammatory intracellular milieu. Importantly, cART treatment restored cGAS and STING expression to levels approaching those of the control group (Figure 6A,F,G), indicating partial normalization of cytosolic DNA‐sensing pathway activation. In addition, we observed that HIV‐1 infection elicited a significant upregulation of IFI6 in hCOs, reflecting enhanced interferon‐stimulated antiviral responses and increased RNA helicase‐associated activity linked to viral replication (Figure 6H). Collectively, these findings support the notion that HIV‐1 triggers robust innate immune activation through both DNA‐sensing and interferon‐stimulated pathways, whereas cART effectively attenuates, but does not fully abolish, these inflammatory responses.

Dot plot analysis of single‐nucleus RNA‐seq data from HIV‐1‐infected hCOs revealed cell‐type specific activation of innate immune and inflammatory pathways. Microglia exhibited the highest expression of inflammatory regulators, including CEBPB, CSF1, CXCL2, IL18, NFKB1, NFKB2, and MAP2K3, consistent with preferential infection and immune activation. Astrocytes and oligodendrocytes showed moderate induction, whereas neurons displayed lower expression, indicating secondary involvement (Figure 6I).

### 2.8 cART Attenuates HIV‐1‐Induced Mitochondrial Damage and Metabolic Dysregulation in hCOs

Single‐nucleus RNA‐seq data revealed an upregulation of several mitochondrial genes during HIV‐1 infection, including three key NDUF family members (core subunits of mitochondrial Complex I) and six ATP synthase genes, suggesting heightened mitochondrial activity or stress responses in infected hCOs (Figure 7A). Violin plots showing cell type–specific expression of mitochondrial oxidative phosphorylation genes NDUFA4, NDUFB9, and NDUFS5 in astrocytes, microglia, and oligodendrocytes derived from snRNA‐seq of control, HIV‐1‐hCO's and cART treated. As shown in Figure 7B, HIV infection reduced expression of multiple oxidative phosphorylation components, particularly in astrocytes and oligodendrocytes, indicating mitochondrial dysfunction. cART treatment partially restored expression of these genes, suggesting recovery of mitochondrial metabolic activity in specific glial populations. Despite this apparent upregulation of mitochondrial gene expression, we observed a significant downregulation of PGC1α, the transcriptional coactivator central to mitochondrial biogenesis and oxidative metabolism (Figure 7C,D).

**FIGURE 7:**
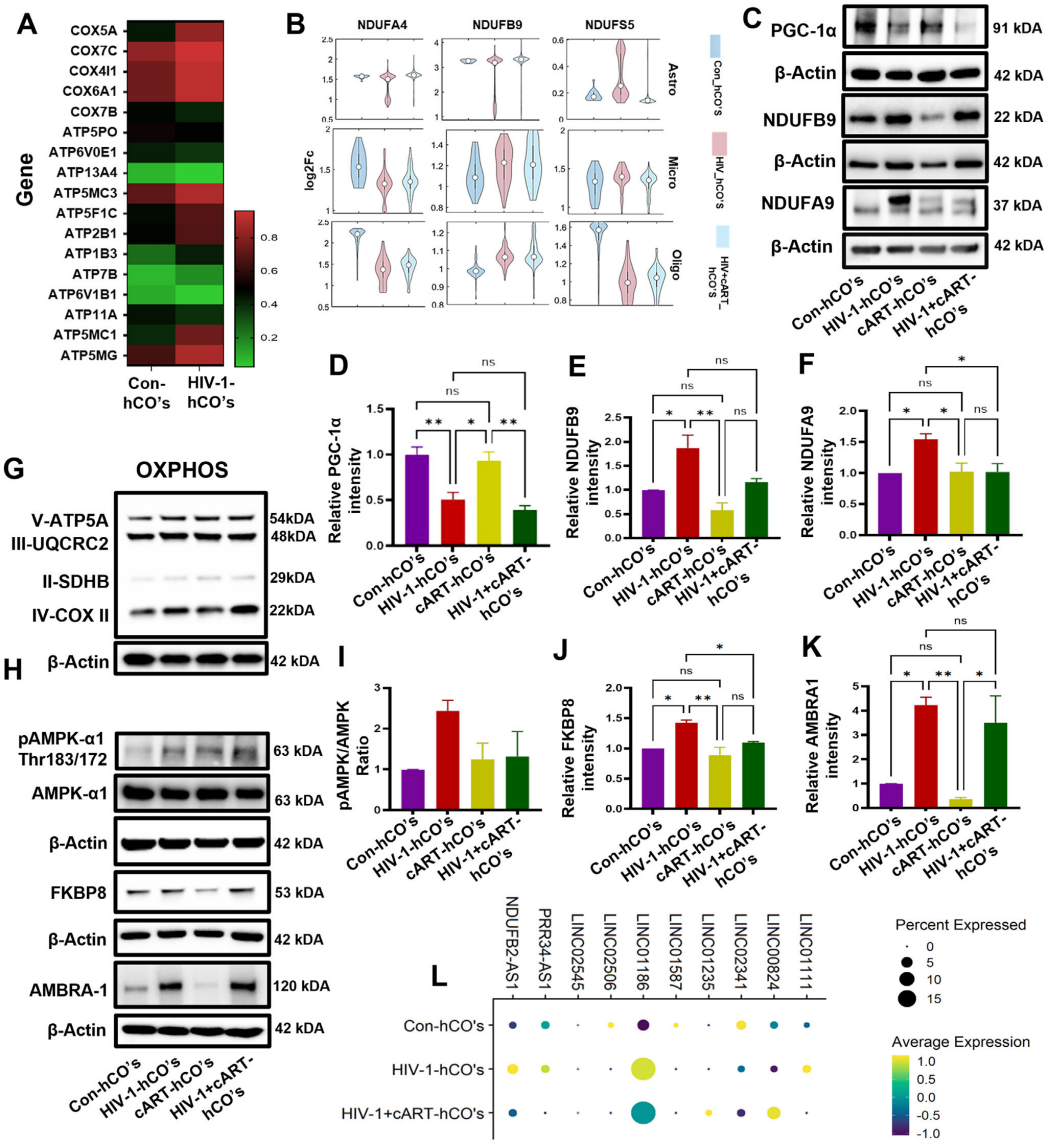
Mitochondrial complex protein and oxidative phosphorylation analysis in HIV‐1‐infected hCOs with and without cART. (A) Complex of V‐related genes and ATP synthase subunit genes were analyzed using single‐nuclei RNA seq using hCOs. (B) Integrated multi‐omics analysis identified the expression of NDUFA4, NDUFB9, and NDUFS5 across multiple cell types, including astrocytes, microglia, and oligodendrocytes. The heatmap depicts the log_2_ fold‐change (HIV‐1 hCO / control and cART‐hCO) for each gene. Expression values were log‐transformed, normalized, and averaged across samples per group, and visualized using the R heatmap package. (C) Right panels indicate the Western blot analysis and respective bar graph, (D) PPARGC1α (PGC1α), (E) NDUFB9, (F) NDUFA9, (G) oxidative phosphorylation (OXPHOS) complex proteins, (H) Western blots analysis of AMPK and mitophagy‐related proteins (I) AMPK, (J) FKBP8, (K) AMBRA1 were analyzed from HIV‐1‐infected hCOs, with and without cART treatment. β‐Actin served as an internal control. Data are presented as the mean ± SD of three independent experiments, with statistical significance determined by one‐way ANOVA followed by Tukey's multiple comparison test at ^*^ *p* < 0.05, ^**^ *p* < 0.01, ns‐Not significant. (L) Integrated snRNA‐seq and snATAC‐seq analysis identified expression profiles of selected long intergenic non‐coding RNAs (LINC RNAs) within microglial populations.

Interestingly, protein‐level analysis confirmed elevated expression of NDUFB9 and NDUFA9, indicating mitochondrial Complex I overactivation, a condition linked to reactive oxygen species (ROS) production, cellular stress, and apoptosis in HIV‐1‐infected hCOs (Figure 7E,F). These changes were accompanied by increased levels of phosphorylated AMPKα at Thr172, a metabolic stress sensor activated during energy deficits (Figure 7H,I). Remarkably, cART treatment attenuated the expression of NDUFB9, NDUFA9, and phospho‐AMPKα, suggesting a partial restoration of mitochondrial function and metabolic homeostasis.

To examine whether HIV‐1‐induced alterations in oxidative phosphorylation promote mitophagy, we analyzed the expression levels of FKBP8 and AMBRA1 under untreated and cART‐treated conditions. Both FKBP8 and AMBRA1 proteins were significantly upregulated in HIV‐1–infected hCOs; however, cART treatment did not significantly alter AMBRA1 expression (Figure 7H,J,K) or oxidative phosphorylation levels (Figure 7G).

Similarly, snRNA‐seq data identified several long non‐coding RNAs (lncRNAs) that could serve as therapeutic targets in disease models. Specifically, lncRNAs such as NDUFB2‐AS1, PRR34‐AS1, LINC02545, LINC02506, LINC01186, LINC01587, LINC01235, LINC02341, LINC00824, and LINC01111 were identified from HIV‐1‐infected hCOs within the microglia subtypes (Figure 7L) and other cell types, as shown in Figure S10. These lncRNAs are implicated in various biological processes, including mitochondrial functions and glycolytic reprogramming [43], as well as NF‐kB signaling. Given their critical regulatory roles, these factors warrant further investigation and may represent novel therapeutic targets for mitigating HIV‐associated neuropathology alongside cART. LINC01186 and NDUFB9‐AS1 were markedly upregulated in HIV‐1‐infected hCOs, with their expression attenuated by cART. In contrast, LINC00824 and LINC02341 were downregulated following infection, but cART treatment restored these effects (Figure 7L). Recent studies demonstrated that TGF‐β/SMAD3 directs LINC01186 to promote epithelial‐mesenchymal transition in lung cancer [44]. Our findings suggest that HIV‐1‐induced dysregulation of LINC01186 may similarly disrupt TGF‐β signaling in hCOs.

### 2.9 HIV‐1 Infection Induces Neuronal Dysfunction, Tau Pathology and BIN1 Upregulation: Special Reference to HAND

Integrated multi‐omics analysis identified NEURL1 as a significantly dysregulated gene in HIV‐1‐infected human cerebral organoids (hCOs). Although NEURL1 transcripts were significantly reduced (Figures 3I,J and 5A), protein expression was increased, indicating a discordance between mRNA and protein levels. No significant differences were detected following cART treatment (Figure 8A). In addition, our immunostaining results supported the western blot findings, demonstrating increased NEURL1 expression in HIV‐1‐infected organoids compared with control groups (Figure 8B and Figure S11). This concordance between immunofluorescence and protein‐level quantification further validates the robustness of NEURL1 upregulation in response to HIV‐1 infection, suggesting a consistent alteration in neuronal regulatory pathways across complementary analytical platforms. However, transcriptomic analysis revealed a significant downregulation of NEURL1 following HIV‐1 infection, indicating a potential post‐transcriptional regulatory mechanism or protein‐level compensatory response that may decouple mRNA abundance from protein expression under inflammatory conditions.

**FIGURE 8:**
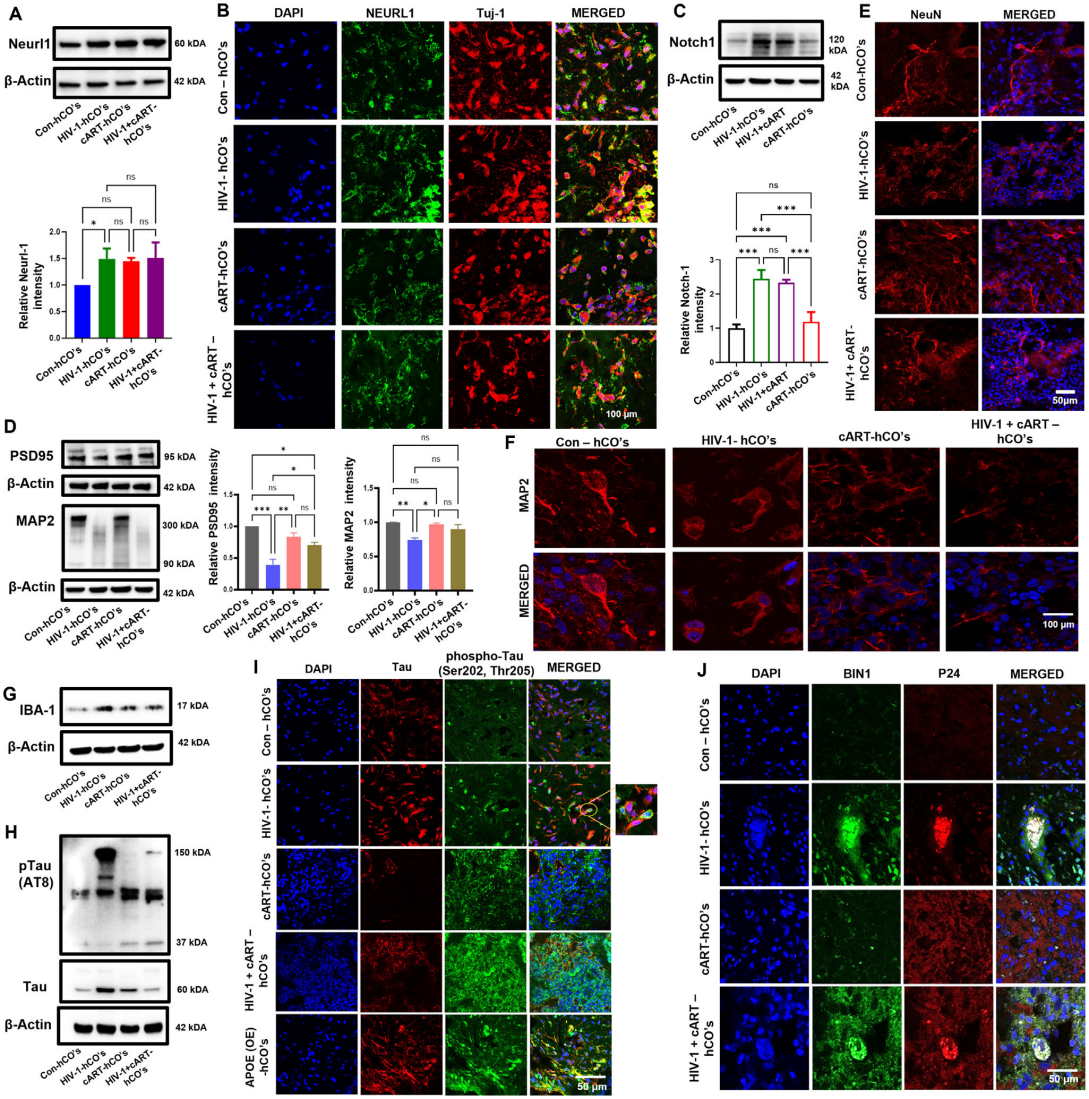
NEURL1‐Mediated Notch signaling modulates PSD95 expression in HIV‐1 infected Human cerebral organoids. (A–E) Western blot analysis showing expression levels of (A) NEURL1 with corresponding bar graph, (B) Immunostaining of NEURL1 expression in cerebral organoid sections using anti‐NEURL1 antibodies (green), β‐Tubulin III (Tuj‐1; red) as a neuronal marker, and DAPI (blue) for nuclear staining. (C) Immunoblot analysis of Notch‐1, and (D) PSD95 and MAP2 in control and HIV‐1‐infected human cerebral organoids (hCOs). β‐actin was used as a loading control. Densitometric quantification is presented as mean ± SD from three independent experiments. Immunostaining of hCOs sections was performed using antibodies against (E) NeuN, and (F) MAP2 (red) in control and HIV‐1‐infected organoids. Nuclei were counterstained with DAPI (blue). Scale bars: 50 µm and 100 µm. Data are presented as the mean ± SD of three independent experiments, with statistical significance determined by one‐way ANOVA followed by Tukey's multiple comparison test at ^*^ *p* < 0.05, ^**^ *p* < 0.01, ^***^ *p* < 0.001, ns‐ Not significant. (G) Representative Western blot and bar graph show the quantification of Iba‐1 levels in hCOs exposed to HIV‐1 infection and cART treatment. (H) Western blot analysis of phospho‐Tau and total Tau protein levels in HIV‐infected organoids. (I) Double immunolabeling was conducted using antibodies against phospho‐Tau (Ser 202 and Thr 205, AT8) (green) and total Tau (red) in HIV‐1_hCOs with or without cART treatment, revealing the presence of hyperphosphorylated Tau. Scale bar = 50 µm. (J) Representative immunofluorescence image showing overexpression of BIN‐1 in HIV‐ infected cerebral brain organoids. Double immunolabeling fluorescence analysis was performed using antibodies against BIN‐1 (green) and HIV‐1 p24 (red) in HIV‐1‐infected hCOs in the presence or absence of cART. Scale bar = 50 µm.

Furthermore, Notch‐1 levels were significantly increased in HIV‐1‐infected organoids compared to controls (Figure 8C). Indeed, to evaluate the functional implications of these molecular changes, we investigated markers of microglial activation, synaptic integrity, and neuronal health. Western blot and immunofluorescence analyses revealed increased Iba‐1 expression, accompanied by marked reductions in PSD95, a postsynaptic density protein essential for synaptic signaling, and MAP2, a dendritic marker, in HIV‐1‐infected organoids (Figure 8D,G). Similarly, the expression of NeuN and MAP2 was decreased in HIV‐infected hCOs compared to cART‐treated groups (Figure 8E,F). Notably, cART treatment partially restored the expression of both PSD95 and MAP2, suggesting that antiretroviral therapy supports healthy neuronal structure and function. Neurons, due to their high energy demands, are particularly susceptible to mitochondrial defects, which promote disease progression via oxidative stress, bioenergetic failure, and apoptosis [45, 46]. Altered NeuN expression patterns are widely used as markers of neuronal injury or loss, offering valuable insight into the underlying pathological processes.

Disease Ontology (DO) enrichment analysis of microglial subtypes in HIV‐1‐infected hCOs revealed significant associations with Alzheimer's disease, neurodegeneration pathways (including Prion and Parkinson's diseases), and the AGE‐RAGE signaling cascade linked to diabetic complications (Figure S12A). As key effectors of the CNS immune response to HIV‐1, the microglia also showed upregulation of several KEGG‐enriched genes, TUBB6, TUBA3C, ATP5MC1, COX5A, NDUFB9, SDHC, PSMA8, DDIT3, AMBRA1, NOX4, and WNT pathway components, suggesting a heightened susceptibility to Alzheimer‐like neuropathogenesis (Figure S12B).

To further explore the neuropathological impact of HIV‐1 infection in the context of HAND, we examined Tau accumulation and BIN1 expression in hCOs. HIV‐1 infection resulted in a significant increase in both total and phosphorylated Tau levels compared with controls, indicating disruption of Tau homeostasis. The accumulation of phosphorylated Tau may promote Tau oligomerization and aggregation, potentially contributing to neuronal dysfunction and neurotoxic outcomes associated with HAND (Figure 8H). Similarly, immunofluorescence staining revealed increased accumulation of phospho‐Tau, BIN1, and HIV‐1 p24 in HIV‐1‐infected hCOs compared with controls (Figure 8I,J). This suggests a possible link between HIV‐1‐induced molecular changes and Tau‐related pathology. Since BIN1 can alter intracellular Tau dynamics, its upregulation may contribute to neuronal damage, increased neuroinflammation, and synaptic dysfunction in the context of HIV [47]. Treatment with cART significantly reduced Tau accumulation and oligomerization, and restored BIN1 levels. These results are consistent with previous studies that suggested HIV‐1 infection as a potential risk factor for neurodegeneration and cognitive decline, including the acceleration of AD‐like pathology in the brain [48, 49].

### 2.10 Single‐Nuclei RNA Sequencing Reveals Transcriptional Alterations in SIV‐Infected Rhesus Macaques Following cART Treatment

For cross‐species validation of the hCO's data, we reanalyzed previously published snRNA‐seq datasets from healthy controls and SIV‐infected, cART‐treated rhesus macaques brain region as described in the Materials and Methods (Figure 9A) [32, 50]. The UMAP projection of aggregated cells is shown in Figure 9B. The major cell populations identified include astrocytes, cerebellar glutamatergic neurons, choroid plexus epithelial cells, cortical projection neurons, cortical pyramidal neurons, GABAergic neurons, glutamatergic neurons, mature oligodendrocytes, microglia, myelinating oligodendrocytes, neurons, and OPCs (Figure 9C). The canonical markers used for cell type annotation are described in Figure 9D. Similar to findings in HIV‐1‐infected hCOs, snRNA‐seq data from the brains of SIV‐infected, cART‐treated RMs brain region revealed considerable changes in cell population composition compared to healthy controls.

**FIGURE 9:**
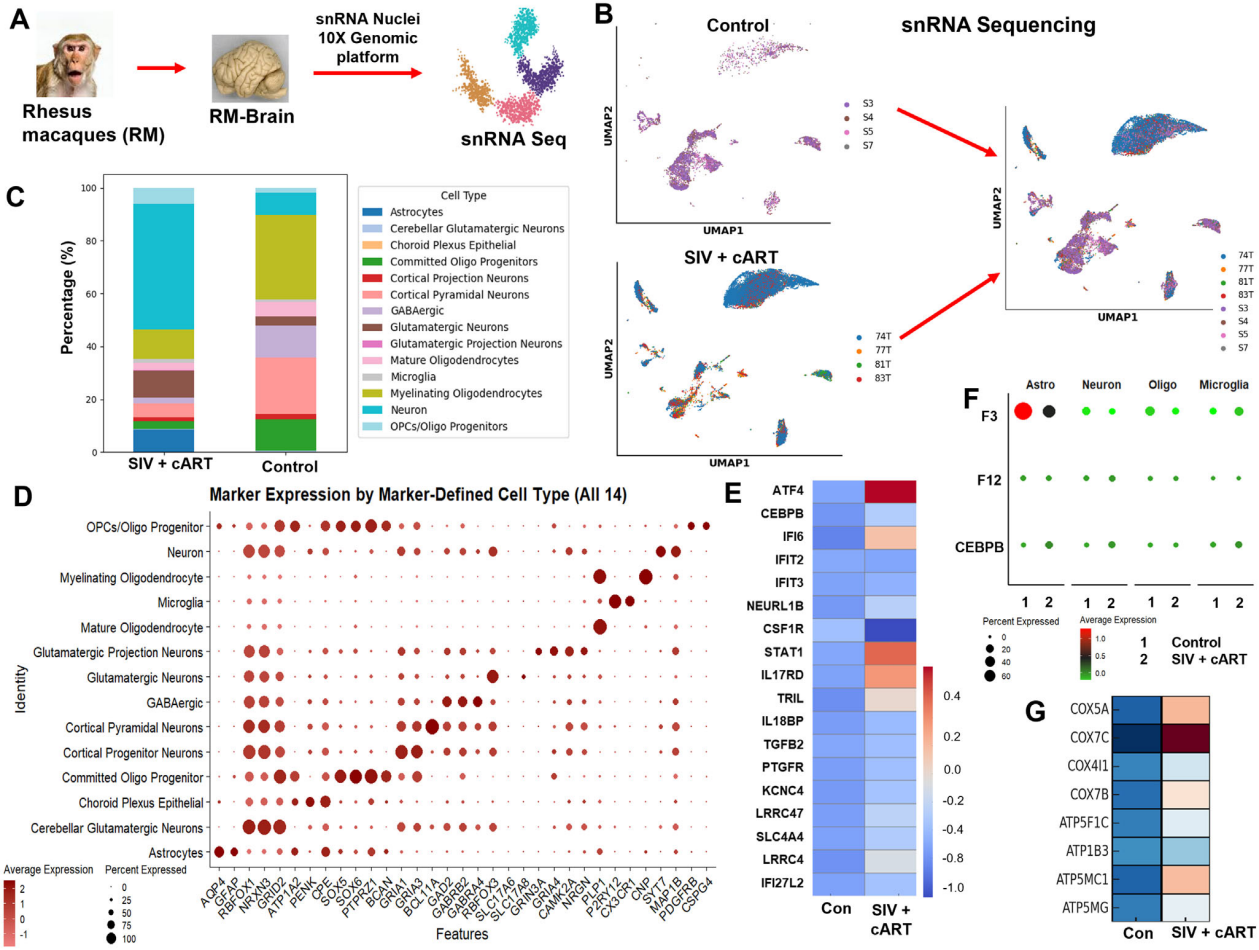
Single‐Nuclei transcriptomic profiling and cell type annotation in a macaque model of SIV infection. (A) Schema of the study design. (B) UMAP plots illustrating transcriptional clustering of cells colored by annotated cell types. (C) Bar plot showing the proportion of identified cell types in SIV‐infected, cART‐treated macaques and healthy controls. (D) Dot plot summarizing the expression of representative marker genes across clusters used for cell type annotations. The diameter of the dot corresponds to the proportion of cells expressing the indicated gene, and the density of the dot corresponds to the average expression across all cell types. (E) A curated panel of immune and inflammation‐associated genes of interest, from control and SIV+cART‐treated RMs. Log‐transformed, normalized expression values were averaged across samples per group and visualized using the heatmap R package. Row‐wise scaling was applied, and a diverging color scheme (blue to red) highlights differential expression. (F) Expression profile of F3, F12, and CEBPB in astrocytes, neurons, oligodendrocytes, and microglia in control vs. SIV‐infected, cART‐treated macaques. (G) Heatmap depicting normalized gene expression of mitochondrial and ATPase‐related genes in the brain from control vs. SIV‐infected and cART‐treated macaques.

Next, we performed cell type‐specific DEG analysis of snRNA‐seq data from SIV‐infected cART‐treated RMs brains compared to uninfected controls from the database to validate the DEGs and predicted altered signaling pathways observed in the hCO's (Figure S13A–D). The analysis was focused on immune‐related pathways, including chemokine signaling, TGF‐β signaling, Type I interferon signaling, and oxidative phosphorylation pathways (Figure S13E–H). Additionally, pathways associated with immune responses, inflammatory responses, synaptic regulation, and the cell cycle are enriched across major cell types, as shown in Figure S14A–D. These findings parallel our observations in hCOs, reinforcing the presence of shared molecular disruptions in both human and macaque models of HIV/SIV neuropathogenesis.

To complement our hCOs data, we also examined the normalized gene expression profile of control and SIV‐infected macaques receiving cART. Heatmap analysis revealed distinct transcriptional alterations, including several stress‐ and immune‐related genes. Notably, ATF4 and STAT1, key regulators of cellular stress responses and interferon signaling, were strongly induced in macaques brain. Interferon‐stimulated genes, including IFI6, also showed elevated expression, reflecting persistent antiviral and inflammatory signaling. Pro‐inflammatory and immune‐regulatory genes, such as CEBPB, IL17RD, and IL18BP, were moderately upregulated in the SIV + cART group, indicating ongoing immune activation (Figure 9E). Furthermore, SIV‐infected, cART‐treated rhesus macaques brain transcriptomics demonstrated residual microglial activation, confirming persistence of neuroinflammation following cART (Figure 9F). Consistent with our hCOs data, the brain of SIV‐infected, cART‐treated macaques brain region showed an upregulation of multiple Complex I subunits and ATP synthase genes relative to uninfected controls (Figure 9G).

## 3 Discussion

HIV‐associated neuroinflammation remains a major contributor to cognitive decline despite effective cART. The molecular pathways linking viral infection, glial activation, and neuronal dysfunction remain incompletely understood. In this study, we establish the IL‐17A/CEBPB signaling and NEURL1‐Notch pathway as underappreciated regulators of HIV‐induced CNS dysfunction. Using human cerebral organoids (hCOs) containing microglia, along with cross‐species validation in SIV‐infected, ART‐treated rhesus macaques, we delineate a mechanistic framework linking microglial activation, cytokine induction, synaptic impairment, mitochondrial dysfunction, and Tau pathology. Our findings highlight early‐stage molecular mechanisms that sustain CNS injury in HIV‐1 infection and demonstrate that cART can partially reverse key inflammatory and metabolic perturbations, underscoring actionable therapeutic targets.

HIV‐induced neuroinflammation begins with microglial activation, which rapidly propagates to astrocytes, neurons, and oligodendrocytes. This disruption of multicellular signaling networks dismantles homeostatic signaling networks, leading to neuronal dysfunction and progressive cognitive decline. Although cell‐type‐specific abnormalities have been documented, the integrated signaling cascades linking these effects to clinical outcomes remain poorly defined. To address this gap, we introduced HIV‐1‐infected iPSC‐derived microglia into mature (60‐day‐old) cerebral organoids using a surface‐seeding strategy. This approach allowed us to investigate microglia‐mediated immune modulation within a physiologically mature neural environment. By focusing on the role of peripherally infected cells in seeding the brain reservoir [51, 52, 53], in the recent model achieves high biological relevance and closely approximates the actual pathophysiology of HIV in humans [54].

Integration of snRNA‐seq and snATAC‐seq, together with publicly available datasets from macaque brains [32, 50] provided a single‐cell resolution of autonomous and intercellular responses. This approach supported mapping cell composition, infection‐ and therapy‐dependent transcriptional programs, developmental trajectories, chromatin accessibility, and active regulatory elements. Notably, cART mitigated many HIV‐driven perturbations, particularly within glia–neuron networks, suggesting mechanisms by which therapy preserves CNS homeostasis.

HIV‐1‐infected microglia exhibited a progressive increase in intracellular p24 antigen by day 6 postinfection, indicating active viral replication. The increased p24 expression was followed by strong upregulation of pro‐inflammatory cytokines (IL‐1β, TNF‐α, IL‐6) and chemokines, including CXCL10 and MCP‐1, consistent with previous reports of HIV‐mediated neuronal injury [55]. Our hCOs recapitulated these features, exhibiting pronounced induction of CXCL10, CEBPB, and IFN‐γ following HIV‐1 infection. IFN‐γ, in turn, activated canonical JAK/STAT signaling by phosphorylating STAT1 and driving its nuclear translocation, thereby driving ISG expression [56]. Importantly, cART treatment reduced STAT1 phosphorylation and suppressed downstream cytokine and chemokine expression, highlighting its therapeutic role in mitigating HIV‐1–induced neuroinflammatory cascades.

The transcription factor CEBPB emerged as a central regulator of HIV‐1‐induced neuroinflammation. A key mediator of immune and inflammatory gene expression [57], CEBPB was robustly upregulated in both HIV‐1–infected hCOs and SIV‐infected, ART‐treated macaque brains. CEBPB is modulated by IL‐17A, a Th17‐derived cytokine that engages NF‐κB and MAPK signaling through its SEFIR domain [58]. It is important to note that, according to the current guidelines, ART should be initiated immediately for all individuals with HIV following confirmed diagnosis [59]. However, cART alone does not completely reverse HIV‐associated neuroinflammation [60, 61, 62, 63]. Overall, findings from the current study support the persistence of neuroinflammation in SIV‐infected, ART‐treated RMs compared with uninfected controls, consistent with observations of hCOs.

Furthermore, similar to findings from hCOs and macaque models, Borjabad et al. demonstrated that STAT1 remains upregulated in postmortem brain tissues from PWH with or without cART, who are affected by HAND [64]. Nguyen et al., also identified STAT1 as a biomarker of HAND using transcriptomic data from postmortem brain tissue [65]. Together, these findings support our observations in the SIV+ART macaque model and hCOs system. Additionally, Canchi et al. demonstrated by immunostaining that, in the prefrontal cortex of postmortem brain tissues, C/EBPβ levels were significantly reduced in neurons and increased in astroglia in HAND patients compared to cognitively normal controls [66].

This IL‐17A/CEBPB signaling has been implicated in autoimmune neuroinflammatory conditions, such as experimental autoimmune encephalomyelitis [9]. Previous studies demonstrate that HIV‐1 activates the complement C3 promoter through NF‐κB‐mediated upregulation of IL‐6, enhancing inflammatory processes associated with HAND [67]. Because HAND is a chronic and progressive disorder, the 9‐day HIV infection paradigm used in this study may not fully capture the cumulative effects of sustained neuroinflammation and neuronal damage observed in vivo. Nevertheless, this model provides valuable insight into the early neuroinflammatory and transcriptional events triggered by HIV infection.

Emerging evidence suggests that IL‐17A enhances glial reactivity, whereas CEBPB integrates JAK/STAT and interferon signaling pathways, thereby promoting sustained immune dysregulation [68]. Dysregulated CEBPB expression may therefore contribute to sustained neuroinflammation in HIV‐1‐infected individuals, even under effective cART. Consistent with this, HIV‐1‐induced NF‐κB activation also upregulates CSF1R, which remains persistently expressed in the brains of virally suppressed SIV‐ and HIV‐infected individuals, indicating that CSF1‐mediated inflammation persists despite viral suppression [69, 70]. Consistent with these findings, CSF1R expression remained elevated in our brain organoid model following cART‐mediated viral suppression, suggesting that microglia may adopt either a protective (M2) or pro‐inflammatory (M1) phenotype depending on disease context [71]. In chronic neurodegeneration, such as HIV infection, the shift toward a protective M2 state appears impaired, contributing to sustained neuroinflammation and neuronal injury.

Notably, NEURL1, a neuronal E3 ubiquitin ligase involved in synaptic stability, was downregulated under these inflammatory conditions, suggesting that microglial CSF1R activation may indirectly influence neuronal homeostasis. Loss of NEURL1 may further amplify IL‐17A/STAT1 signaling and destabilize synaptic networks, consistent with studies demonstrating that NEURL1 deficiency impairs memory formation and neuronal function [72]. Although a direct mechanistic relationship between NEURL1 and CEBPB has not been established, our findings highlight early‐stage molecular connections that sustain CNS injury in HIV‐1 and demonstrate that cART can partially reverse critical inflammatory and metabolic perturbations, underscoring actionable therapeutic targets. Furthermore, we identify a striking divergence between NEURL1 transcript and protein levels following HIV‐1 infection, where NEURL1 mRNA is significantly downregulated while protein expression is concurrently elevated. This discordance suggests a complex layer of post‐transcriptional and post‐translational regulation, potentially driven by infection‐induced changes in protein stability, translational efficiency, or microglia–neuron signaling dynamics. Together, these findings emphasize that HIV‐1‐associated neuroimmune activation is governed not only by transcriptional reprogramming but also by multilevel regulatory mechanisms that fine‐tune the disruption of neuronal pathways in the context of chronic viral exposure. Similar transcript–protein discrepancy has been reported in other disease contexts; for instance, Teider et al. (2010) demonstrated reduced NEURL1 transcript levels in primary medulloblastoma tumors and cell lines [ 73 ], highlighting the context‐dependent and multilayered regulation of NEURL1 under pathological conditions. We acknowledge that further mechanistic studies are required to define the relationship between NEURL1, CEBPB, and HIV‐associated neuroinflammatory pathways.

Importantly, cART treatment restored NEURL1 gene expression and improved the levels of key synaptic markers, including PSD95 and MAP2, indicating partial recovery of neuronal homeostasis, although some HIV‐associated neuroinflammatory and molecular abnormalities persisted. Collectively, persistent CSF1R signaling and altered NEURL1 expression contribute to the dysregulated neuroimmune environment in HIV‐associated CNS injury, positioning NEURL1 as a key integrator of inflammatory signaling and synaptic integrity with therapeutic potential for HAND. Concordant with neuropathology in other neurodegenerative diseases, such as Alzheimer's and Parkinson's, HIV‐1‐infected hCOs exhibited pronounced mitochondrial dysfunction [74, 75].

HIV‐1 infection was associated with altered expression of mitochondrial electron transport chain (ETC) genes, including NDUFB9 and NDUFA9, accompanied by reduced expression of the mitochondrial biogenesis regulator PGC1α [43, 76]. These changes likely represent a compensatory transcriptional response to cellular energetic stress and mitochondrial remodeling rather than direct evidence of enhanced oxidative phosphorylation. Consistent with this interpretation, mitochondrial dysfunction was further supported by increased ROS production, dysregulated expression of ETC Complex I subunits (NDUFB9 and NDUFA9), and activation of apoptotic pathways [43]. Importantly, cART treatment reduced CEBPB expression, restored PGC1α levels, improved ETC‐associated gene expression, and decreased ROS accumulation, collectively confirming the interconnected roles of inflammatory signaling and mitochondrial homeostasis. Notably, CEBPB also regulates APOE expression and preferentially promotes the ApoE4 isoform, a well‐established genetic risk factor for Alzheimer's disease [77]. Together, these findings suggest that CEBPB upregulation not only initiates HIV‐induced inflammatory and metabolic stress but may also intersect with neurodegenerative disease‐associated pathways, linking HIV‐associated neuroinflammation to broader mechanisms of cognitive decline.

Tau pathology further underscores the convergence of HIV‐1 infection and neurodegeneration. Inflammatory mediators and viral proteins can promote Tau phosphorylation, which contributes to synaptic dysfunction and HAND‐like pathology [78]. The adaptor protein BIN1, a Tau interactor, links Tau phosphorylation to calcium homeostasis and synaptic regulation [79, 80]. In our study, cART treatment reduced Tau aggregation in HIV‐1‐infected hCOs, consistent with reports that nucleoside reverse transcriptase inhibitors such as lamivudine (3TC) mitigate Tau‐driven neurodegeneration [81]. Taken together, these findings suggest that antiretroviral therapy may confer direct neuroprotective effects beyond viral suppression.

Consistent with the limitations of experimental design, our observations primarily reflect early and subacute neuroinflammatory and metabolic responses to HIV‐1 infection rather than chronic disease progression. The prophylactic cART paradigm, in which ATRIPLA was administered prior to viral exposure, likely contributed to the observed partial normalization of inflammatory, oxidative stress, and mitochondrial dysfunction markers and may not fully recapitulate clinical HAND, where treatment is initiated after established infection. Similarly, the 9‐day infection period in hCOs captures acute‐to‐subacute molecular and cellular responses, including cytokine induction, glial activation, mitochondrial stress, and early synaptic regulatory changes, but does not model long‐term processes such as viral persistence, latency, or progressive neurodegeneration.

Accordingly, the transcriptional, proteomic, and signaling changes observed in this study, including IL‐17A/CEBPB activation, cGAS–STING signaling, oxidative stress pathways, and NEURL1‐associated neuronal regulation, are best interpreted as early mechanistic events that initiate or prime downstream neuroimmune dysregulation rather than as fully established or irreversible pathology. This framework may underestimate sustained glial activation, cumulative neuronal loss, and progressive synaptic dysfunction characteristic of chronic HIV neuropathology. Future studies incorporating post‐infection cART initiation and extended infection durations (30–60 days or longer) will be essential to better model chronic disease and define the persistence or reversibility of these pathways.

In summary, our findings provide a comprehensive multi‐omic characterization of early HIV‐1‐associated neuroinflammatory and neurodegenerative signatures in human cerebral organoids containing integrated microglia, complemented by cross‐species validation in SIV‐infected, cART‐treated rhesus macaques. HIV‐1 infection induced extensive cell‐type‐specific transcriptional reprogramming across neuronal and glial populations, characterized by coordinated activation of inflammatory, interferon‐responsive, oxidative stress, and mitochondrial dysfunction pathways. We identified IL‐17A/CEBPB signaling and the NEURL1–Notch axis as key molecular networks associated with HIV‐mediated CNS dysfunction, linking microglial activation to synaptic alterations, mitochondrial stress, and Tau‐related pathology (Figure 10). Importantly, HIV‐1 infection was associated with dysregulation of innate immune pathways, including cGAS‐STING and IFI6 signaling, indicative of persistent neuroimmune activation despite antiretroviral treatment. Even though cART partially attenuated several HIV‐induced molecular perturbations, many transcriptional and signaling abnormalities remained evident, suggesting incomplete restoration of CNS homeostasis. Furthermore, the discrepancy between NEURL1 transcript and protein expression highlights the complexity of regulatory mechanisms underlying HIV‐associated neuronal dysfunction and warrants further investigation. Collectively, these findings elucidate key molecular mechanisms underlying early NeuroHIV pathogenesis and highlight potential therapeutic targets for reducing neuroinflammation, preserving mitochondrial function, and maintaining neuronal integrity.

**FIGURE 10:**
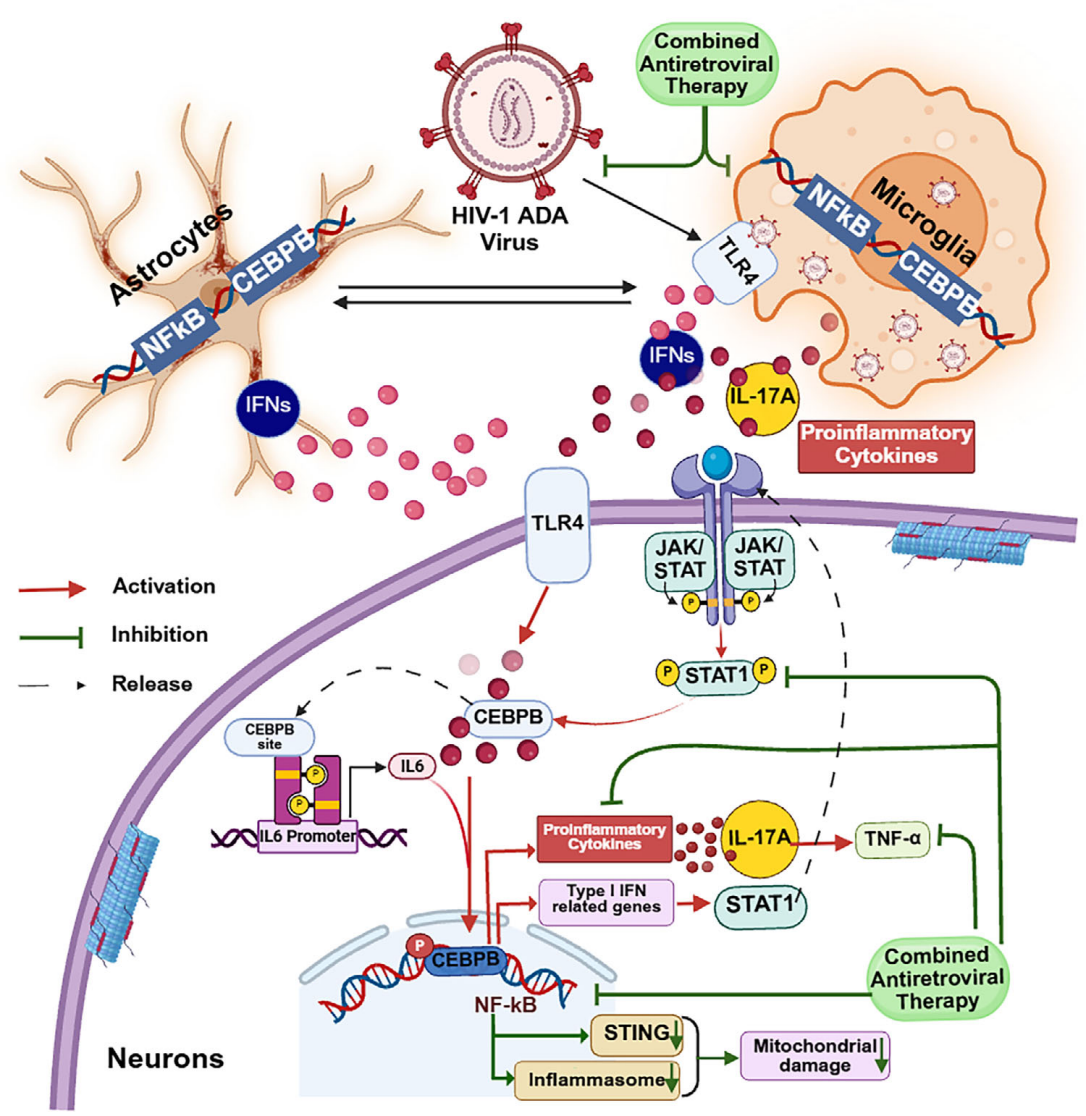
Schematic representation of HIV‐1‐induced neuroinflammatory signaling in microglia, astrocytes, and neurons, and the modulatory effects of cART. HIV‐1 ADA virus infects microglia and astrocytes, triggering TLR4‐mediated activation of transcription factors CEBPB and NF‐κB. In microglia, this leads to the production and release of IL‐17A, interferons, and other proinflammatory cytokines. These inflammatory mediators activate TLR4 and JAK/STAT signaling pathway in neighboring neurons, leading to STAT1 phosphorylation and nuclear translocation. Astrocytes also contribute to the inflammatory milieu through CEBPB and NF‐κB signaling and IFN release. IL‐17A and TNF‐α enhance inflammatory signaling cascades, perpetuating neuronal dysfunction. Combined antiretroviral therapy (cART) attenuates HIV‐1 infection and inhibits downstream inflammatory signaling, including the release of IL‐17A, TNF‐α, and activation of NF‐κB and STAT1. Arrows indicate activation (red), inhibition (green), and release or secretion (dashed black).

## 4 Materials and Methods

### 4.1 Cell Culture

The human induced pluripotent stem cell (iPSC) line (Episomal, PBMC‐derived; Alstem, Cat. #iPS15), hereafter referred to as iPS15, was maintained in feeder‐free conditions using mTeSR1 Plus medium (STEMCELL Technologies, Cat. #85850) supplemented with ROCK inhibitor (Y‐27632; STEMCELL Technologies, Cat. #72307). Cells were cultured on Matrigel‐coated 6‐well plates, with medium changes performed every other day. Cultures were incubated at 37°C in a humidified atmosphere containing 5% CO_2_.

### 4.2 Cerebral Organoid Development

Human cerebral organoids were generated using the human iPSC line (iPS15) and the STEMdiff Cerebral Organoid Kit (STEMCELL Technologies, Cat. #08570), following the manufacturer's instructions. Freshly prepared media were used for each developmental stage: embryoid body (EB) formation (Day 0–5), neural induction (Day 5–7), expansion (Day 7–10), and maturation (Day 10–90). On Day 0, iPSCs were dissociated using Gentle Cell Dissociation Reagent (STEMCELL Technologies, Cat. #100‐0485) and resuspended in mTeSR medium (STEMCELL Technologies, Cat. #100‐0276). Cells were centrifuged at 300 × g for 5 min, and the pellet was resuspended in EB seeding medium containing 10 µM Y‐27632. Approximately 9000 cells per well were seeded into a low‐attachment 96‐well plate for EB formation and incubated at 37°C with 5% CO_2_.

Days 2 and 4, 100 µL of EB formation medium was added to each well. On Day 5, 1–2 EBs per well were transferred into a low‐attachment 24‐well plate containing induction medium and incubated at 37°C with 5% CO_2_. On Day 7, EBs were embedded in 15 µL of Matrigel (Corning, Cat. #354‐277) per EB on an organoid embedding sheet. A total of 4–6 embedded EBs were placed in each well of an ultra‐low‐attachment 6‐well plate containing 3 mL of expansion medium and incubated at 37°C with 5% CO_2_.

On Day 10, the expansion medium was replaced with maturation medium, and organoids were transferred to an orbital shaker and maintained at 37°C with 5% CO_2_. Half‐media changes were performed every 3–4 days until the experiments were done, as illustrated in Figure 1a.

### 4.3 Derivation of Microglia From iPSC Cells

Hematopoietic progenitor cells (HPCs) were derived from the iPS15 line using the STEMdiff Hematopoietic Kit (STEMCELL Technologies, Cat. #05310) according to the manufacturer's instructions. The HPCs were then seeded and differentiated into microglia using the STEMdiff Microglial Differentiation Kit (Cat. #100‐0019), followed by further maturation with the STEMdiff Microglial Maturation Kit (Cat. #100‐0020), as per the manufacturer's protocol. Briefly, hiPSC‐derived HSCs (2 × 10^5^ cells/well) were plated in Matrigel‐coated 6‐well plates containing 2 mL of STEMdiff Microglia differentiation medium and maintained at 37°C with 5% CO_2_. Cell cultures were refreshed every other day by supplementing with 1 mL of STEMdiff Microglia differentiation medium for 12 days. Following this initial period, the entire cell suspension was centrifuged at 300 g for 5 min and subsequently seeded into fresh Matrigel‐coated 6‐well plates with 1 mL of STEMdiff Microglia differentiation medium, maintaining the same culture conditions for an additional 12 days with regular medium replenishment. On day 24, 1 × 10^6^ cells (at a density of 1 × 10^5^ cells/cm^2^) were harvested, centrifuged at 300 g for 5 min, and cultured in Matrigel‐coated plates with STEMdiff Microglia maturation medium for 4 days. On day 28, the hiPSC‐derived induced iMGs were collected for subsequent HIV infection.

### 4.4 HIV‐1 ADA Infection and cART Treatment of hCOs

The R5‐tropic HIV‐1 ADA strain was propagated in human PBMC or MDMs and titrated as described previously [82], and used for infecting hCOs. iPSC‐derived microglia (5 × 10^4^ cells; i.e., 50 000 cells/organoid) were cultured in 6‐well plates and infected with HIV‐1 ADA at a multiplicity of infection (MOI) of 0.5 in microglial maturation medium supplemented with Polybrene (5 µg/mL) to enhance viral entry. After 12–16 h of incubation, the cells were washed with PBS and maintained in fresh maturation medium for 24 h. The HIV‐infected microglia were then surface‐seeded onto 60‐day‐old cerebral organoids and co‐cultured for an additional 9 days to allow integration and interaction within the human cerebral brain organoids. The media was partially replaced every 3 days.

In parallel, 60‐day‐old organoids were pre‐treated for 2 h with ATRIPLA, a combination antiretroviral therapy (cART) regimen containing 5 µM each of Efavirenz (EFV), Emtricitabine (FTC), and Tenofovir (TDF), with or without subsequent HIV‐1 ADA infection, and maintained for 9 days. Culture supernatants were collected daily, and HIV‐1 p24 antigen levels were quantified using ELISA on days 1, 3, 6, and 9 postinfections.

### 4.5 snRNA‐Seq Data Analysis of Rhesus Macaque Brain Region

No animal experiments were performed as part of this study. All analyses were conducted using publicly available snRNA‐seq data sets of healthy control macaque and SIV‐infected, cART‐treated macaque brain region that were previously published [32, 50]. The snRNA‐seq data of the healthy control rhesus macaques (RMs) were obtained from NCBI GEO, accession # GSE233278, and the data of the SIV‐infected, ART‐treated RMs were obtained from NCBI GEO, accession # GSE209606 [32, 50].

As described in the original publication [32], four RMs were infected with 200 TCID50 of SIVmac251 virus. Five weeks after infection, the animals received daily subcutaneous injections of antiretroviral therapy (ART) consisting of emtricitabine (FTC: 40 mg/mL), tenofovir (TFV: 20 mg/mL) and dolutegravir (DTG: 2.5 mg/mL) administered at 1 mL/kg body weight. Following 30 weeks of cART, the animals were perfused with phosphate‐buffered saline (PBS) containing 1 U/mL heparin to remove blood from the brain, after which brain tissues were harvested, snap‐frozen in liquid nitrogen, and processed for snRNA‐seq analysis as previously described [32].

### 4.6 Single‐Nucleus Dissociation and Library Preparation

For single‐nucleus ATAC sequencing (snATAC‐seq), nuclei were isolated using Nuclei Extraction Buffer (130‐128‐024, Miltenyi Biotec) supplemented with a protease inhibitor cocktail (5892791001; Roche). Human cerebral organoids were finely chopped into fragments smaller than 0.5 mm and homogenized in 2 mL of ice‐cold lysis buffer using a Dounce homogenizer. The homogenate was incubated on ice for 5 min, followed by adding another 2 mL of lysis buffer. The lysate was passed through a 40‐µm cell strainer and centrifuged at 500 × *g* for 5 min at 4°C. The pellet was resuspended in fresh lysis buffer, washed with 4 mL, and incubated on ice for 5 min. After a second centrifugation step, the nuclei pellet was resuspended in Nuclei Buffer (10x Genomics, PN‐2000153), filtered through a 5‐µm cell strainer, and counted.

For single‐nucleus RNA sequencing (snRNA‐seq), RNase inhibitors (Promega, N2615; Life Technologies, AM2696) were added to the lysis buffer to preserve RNA integrity. The final nuclei pellet was resuspended in Nuclei Suspension Buffer (1× PBS, 1% bovine serum albumin, 0.1% RNase inhibitor) and stored in CryoStor CS10 freezing media. Libraries for both snATAC‐seq and snRNA‐seq were prepared using the 10x Genomics Chromium platform according to the manufacturer's protocol.

### 4.7 Single‐Cell RNA and ATAC Sequencing

#### 4.7.1 Cell Thawing and Nuclei Isolation

Cryopreserved cells were thawed following the CryoStor CS10 Thawing Protocol (StemCell Technologies, https://tinyurl.com/veyk9ab5) using pre‐warmed RPMI + 10% FBS for the initial 1:10 dilution and 1X PBS + 0.04% for the washing and final resuspension steps. Thawed cells were stained with acridine orange and propidium iodide (Logos Biosystems PN‐F23001) and assessed for viability, concentration, and singleness using the LUNA‐FX7 Dual Fluorescence Cell Counter (Logos Biosystems). Nuclei were isolated following the 10x Genomics Low Cell Input Nuclei Isolation Protocol (PN‐CG000365 Rev B, http://tinyurl.com/ycynrdhj). Approximately 40 000 pelleted cells per sample were resuspended in 1X Lysis Buffer, incubated on ice for 3 min, washed twice, and resuspended in a proprietary buffer. The nuclei were again assessed for viability, concentration, and singleness using the LUNA‐FX7 [83].

### 4.8 10x Genomics Multiome Assay for Dual Chromatin Accessibility and Gene Expression Profiling

Multiome library preparation was completed using the 10x Genomics Chromium X Controller and the Chromium Next GEM Single Cell Multiome ATAC + Gene Expression Reagent Bundle (10x Genomics PN‐1000283) following the manufacturer's user guide (PN‐CG000338 Rev F, http://tinyurl.com/bdhe7rs4). Freshly isolated nuclei suspensions were incubated in a transposition mix to simultaneously fragment open regions of chromatin and ligate adapter sequences to the ends of the fragmented DNA. Approximately 16 500 nuclei were transposed per sample, with an ultimate recovery target of 10,000 nuclei per sample. Transposed nuclei, a master mix of reverse transcription and template‐switching reagents, and gel beads coated with barcoding oligos were then microfluidically mixed into an oil medium to form gel beads in emulsion (GEMs) using the Chromium Controller instrument (10x Genomics). Individual partitioned single‐nucleus reaction mixtures were formed by lysing the cells and dissolving the gel beads within the GEMs. The GEMs were incubated to generate transposed DNA fragments and full‐length cDNA, both tagged with cell barcodes, unique molecular identifiers (UMIs), and 5’ and 3’ priming sites provided by the gel bead oligos. A quenching agent was added to stop the reaction, and GEMs were stored at −80°C for up to 4 weeks. The GEMs were thawed, broken, and purified using magnetic DNA‐binding beads (Dynabeads MyOne SILANE beads, Invitrogen). The purified products were pre‐amplified via PCR (7 cycles) with primer pairs specific to the DNA fragments (for ATAC) and cDNA (for Gene Expression). Pre‐amplified products were then split into separate aliquots for ATAC and Gene Expression (GEX) library preparation.

### 4.9 ATAC Library Preparation

The Illumina P7 sequencing adapter and a uniquely identifying single index were added to pre‐amplified, transposed DNA fragments via PCR (8 cycles). The completed ATAC sequencing libraries were then purified using SPRIselect beads, visualized and quantified using the Bioanalyzer High Sensitivity DNA, and pooled together before sequencing.

### 4.10 Gene Expression Library Preparation

The pre‐amplified cDNA was further amplified via PCR (7 cycles) to generate enough mass for library preparation and subsequently purified with SPRIselect beads. The cDNA was subjected to fragmentation, end repair, A‐tailing, double‐sided size selection using SPRIselect beads, and Illumina sequencing adaptor ligation. Uniquely identifiable dual indexes were added via PCR (16 cycles). The completed gene expression libraries were then purified using SPRI Select beads, visualized and quantified using the Bioanalyzer High Sensitivity DNA chip, and pooled together for sequencing.

### 4.11 Sequencing and Data Analysis

ATAC and GEX pools were sequenced separately at the UNC High Throughput Sequencing Facility. Pools were denatured and diluted following standard Illumina protocol and spiked with 1% PhiX sequencing control (Illumina) before sequencing. ATAC libraries were sequenced on two NextSeq 2000 P4 flow cells to a total depth of 3.1 billion read pairs passing quality filters. GEX libraries were sequenced on a single NovaSeq 6000 S4 flow cell to a total depth of 8.0 billion read pairs passing quality filters. Libraries were sequenced in the appropriate format for ATAC (Read 1: 50 cycles, i7 index: 8 cycles, i5 Index: 24 cycles, Read 2: 50 cycles) or GEX (Read 1: 28 cycles, i7 index: 10 cycles, i5 Index: 10 cycles, Read 2: 90 cycles) accordingly. Sequencing data was aligned to 10x's prebuilt human reference genome (GRCh38‐2020‐A, https://tinyurl.com/mwvy8e9m) and processed through Cell Ranger ARC v2.0.2 using default settings.

### 4.12 Single‐Nucleus Multi‐Omics Analysis

For the single‐nucleus multi‐omics analyses (snRNA‐seq/snATAC‐seq), organoids were assigned to three experimental groups: control (Con‐hCOs), HIV‐1 infected (HIV‐1‐hCOs), and HIV‐1 infected with cART treatment (HIV‐1+cART‐hCOs), as well as from rhesus macaque (RM) SIV‐infected animals receiving ART and their corresponding controls. A cART‐only group was not included in the final multi‐omics analysis because no substantial molecular changes were observed compared with control organoids.

#### 4.12.1 Preprocessing and Quality Control

Raw sequencing reads were processed with Cell Ranger ARC v2.0.2 (10x Genomics), aligned to GRCh38, and used to generate feature‐barcode matrices. Downstream analyses were performed using Seurat v5 and Signac v1.14 in R. Seurat objects with separate RNA and ATAC assays were created per sample. EnsDb provided peak annotations for ATAC‐seq*. Hsapiens.v86*. To ensure unique cell IDs, Seurat objects were merged with dataset‐specific prefixes. Quality control filtering used the following thresholds: ATAC‐seq—fragments >200 and <30 000; nucleosome signal <3; TSS enrichment >1. RNA‐seq—UMI counts >1000 and <30 000; genes expressed >500.

For cross‐species validation of the hCO dataset, we reanalyzed publicly available snRNA‐seq data of healthy control and SIV‐infected, ART‐treated macaque brains [32, 50]. Data were imported into R using Zellkonverter [84], and converted into a Seurat object to enable downstream processing. Doublets were removed using the DoubletFinder package in R. Low‐quality nuclei were removed based on three metrics: nuclei with gene count <200 or >10 000, UMI count <400 or >1000, and nuclei with mitochondria UMI proportion greater >1%. As a result, a total of 28,760 and 7217 high‐quality nuclei were retained for downstream analysis in the SIV‐infected ART‐treated and control RMs, respectively. Downstream analysis of RMs data was carried out using the same pipeline used for analyzing the hCO dataset.

#### 4.12.2 Normalization, Integration, and Dimensionality Reduction

For the hCO dataset, RNA‐seq data were normalized using *NormalizeData*, and 2000 highly variable genes were selected using *FindVariableFeatures*. PCA was applied after scaling. Harmony‐based batch correction was performed on PCA embeddings using *IntegrateLayers* (method = “Harmony Integration”). For ATAC‐seq data, peaks were filtered with *FindTopFeatures* (min.cutoff = 5), normalized using *RunTFIDF*, and reduced using SVD. A weighted nearest neighbor (WNN) graph was constructed via *FindMultiModalNeighbors* using Harmony‐integrated RNA (top 30 PCs) and LSI‐reduced ATAC (dimensions 2–40). UMAP embedding was computed using *RunUMAP*.

For the RMs, data normalization was performed using SCTransform within Serat, and batch effects were corrected using Harmony, followed by PCA. The top 20 principal components (PCs) were selected for downstream analysis.

#### 4.12.3 Cell Clustering and Annotation

Unsupervised clustering was performed for both datasets following dimensionality reduction. In the hCO dataset, clustering was performed using the Louvain algorithm via *FindClusters* (resolution = 0.8) on the WNN graph. For the RMs dataset, clustering was performed using the Louvain algorithm implemented via the *FindClusters* function in the Seurat package, with 0.3–0.5 resolution. These unsupervised clustering approaches allowed for the identification of transcriptionally distinct cell populations in the single‐nucleus RNA‐seq dataset.

For both datasets, cluster‐specific marker genes were identified using *FindAllMarkers* (log2FC ≥ 1, min.pct = 0.1, FDR‐adjusted *p* < 0.01). Manual annotation was performed using known cell‐type markers from the literature and public databases [85]. Broad categories included astrocytes (Astro), oligodendrocytes (Oligo), neural stem cells (NSCs), neurons, microglia, oligodendrocyte precursor cells (OPCs), and cycling radial glia cells (cRGCs). To assess cell‐type composition, annotated single‐cell RNA sequencing data were analyzed by counting the number of cells assigned to each cell type within each treatment group. Cell counts were then normalized to the total number of cells in the corresponding treatment group to calculate the relative proportion of each cell type. Thus, the sum of all cell‐type percentages within each treatment group equaled 100%. The resulting proportions were used to evaluate changes in cellular composition across experimental conditions using the *CellPercentagesByCellType* analysis method [86].

#### 4.12.4 Differential Expression, Accessibility, and Pathway Analysis

For the hCO dataset, differential expression (DE) and accessibility (DA) were analyzed per cell type. Comparisons were made between HIV‐1‐hCOs vs. Con‐hCOs and HIV‐1+cART‐hCOs vs. HIV‐1‐hCOs using *FindMarkers* (Wilcoxon test; log2FC ≥ 1; min.pct = 0.05), *p*‐values < 0.05 (Bonferroni) were considered significant. For ATAC‐seq, differentially accessible regions (DARs) were identified using the same criteria. Nearest genes to DARs were assigned using *ClosestFeature*. Functional enrichment was performed using the fgsea R package with Gene Ontology (GO) biological processes from MSigDB v2024.1. Pathways with adjusted *p* < 0.05 and ≥2 genes were considered significant. Leading‐edge genes were extracted, and network plots were generated per cell type to visualize pathway–gene relationships. KEGG pathway gene sets were also used to examine expression trends in pathways of interest. Enrichr analysis was performed using DEGs for microglia.

RMs data, differential gene expression analysis was performed for each cluster using Scanpy's rank genes group's function (Wilcoxon rank‐sum test; adjusted *p*‐value < 0.05 and log fold change > 0.25). To reduce cell‐level variability and improve robustness, pseudobulk gene expression profiles were generated by aggregating cells at the sample level within each cell type. Functional enrichment analysis of biological processes, GO Biological Process pathways, was performed using the same fgsea pipeline as previously described (based on MSigDB v2024.1). Likewise, upregulated genes were mapped from gene symbols to ENTREZ gene IDs using the bitr function from the clusterProfiler R package, using the Bioconductor package with the org.Hs.eg.db database used as the annotation database. KEGG pathway enrichment was then performed using the enrich KEGG function, specifying Homo sapiens (organism = “hsa”) and a significance threshold of *p* < 0.05, following the same criteria and visualization strategy as in the hCOs.

### 4.13 ELISA Assay

**p24 ELISA**–HIV‐1 viral load in supernatants from hCOs was quantified using the HIV‐1 p24 SimpleStep ELISA Kit (Abcam, Cat# ab218268), following the manufacturer's instructions. Supernatants were collected every three days up to 9 days post‐HIV‐1 infection, including during cART treatment. Briefly, 50 µL of sample or standard was added to each well of the ELISA plate, followed by 50 µL of antibody cocktail. The plate was incubated at room temperature for 1 h. Wells were then washed three times with wash buffer. Subsequently, 100 µL of TMB substrate was added to each well and incubated for 10 min in the dark on a shaker at 400 rpm. The reaction was stopped by adding 100 µL of stop solution and incubating for 1 min on the shaker. Absorbance was measured at 450 nm using a microplate reader [87].

### 4.14 Cytokine Quantification by Sandwich ELISA

Supernatants were collected from HIV‐1–infected and cART‐treated hCOs on day 6 post‐infection for cytokine analysis. IL‐6, IL‐1β, and IL‐4 levels were quantified using ELISA kits specific to each cytokine, according to the respective manufacturers’ protocols. Briefly, 50 µL of each sample or standard was added in duplicate to wells of a 96‐well plate pre‐coated with capture antibody. An equal volume (50 µL) of biotin‐conjugated detection antibody was then added to each well, followed by a 2‐h incubation at room temperature. Wells were washed three times with the provided wash buffer. Streptavidin‐HRP conjugate (100 µL) was added to each well and incubated for 30 min at room temperature. After additional washes, TMB substrate solution was added, and the plate was incubated in the dark for 30 min. The reaction was stopped by adding 100 µL of stop solution per well, and absorbance was measured at 450 nm using a microplate reader. Cytokine concentrations were calculated using standard curves generated with known concentrations of each cytokine.

### 4.15 Western Blotting

At the end of the experimental time course, hCOs were harvested and washed twice with ice‐cold phosphate‐buffered saline (PBS) to remove residual media and debris. hCOs were then lysed using RIPA Lysis and Extraction Buffer (Thermo Scientific, Cat# 89901), supplemented with protease and phosphatase inhibitor cocktails according to the manufacturer's instructions. Lysates were clarified by centrifugation at 14 000 × g for 15 min at 4°C, subjected to SDS‐PAGE using 4%–20% gradient gels, and transferred to polyvinylidene difluoride (PVDF) membranes using a Western blotting system (Bio‐Rad). Membranes were blocked with EveryBlot Blocking Buffer (Cat# 12010020, Bio‐Rad) for 10 min, followed by incubation with specific primary antibodies at 4°C overnight. After primary antibody incubation, membranes were washed with TBST and incubated with HRP‐conjugated secondary antibodies at room temperature for 1 h. Protein signals were visualized using the Clarity Max Western ECL Substrate system (Cat# 1705062, Bio‐Rad). Images were acquired and analyzed using ImageJ software (National Institutes of Health, Bethesda, MD, USA). Protein expression levels (band intensities) were normalized to β‐actin and expressed as fold change relative to the control group, as previously described [88]. Details of the primary antibodies used are listed in Table S1.

### 4.16 Immunocytochemistry

The hCOs were fixed in 4% paraformaldehyde overnight at 4°C and washed thrice with PBS. After fixation, the hCOs were dehydrated in 30% sucrose solution at 4°C. Subsequently, the hCOs were embedded in Tissue‐Tek O.C.T. compound (Cat# 4583) and frozen on dry ice. The frozen hCOs were sectioned into 10 µm slices using a cryostat and mounted onto glass microscope slides. The sections were blocked with blocking buffers containing 4% normal donkey serum (Cat# S‐30‐M) and 0.1% Triton X‐100 in 1× PBS for 1 h at room temperature. Next, the sections were incubated overnight at 4°C with primary antibodies (1:500 dilution, listed in Table S2) diluted in blocking buffer without Triton X‐100. After three washes with PBS, the sections were incubated for 1 h at room temperature in a light‐protected humidified chamber with secondary antibodies diluted 1:1000: Alexa Fluor 488 (Donkey Anti‐Mouse), Alexa Fluor 488 (Donkey Anti‐Rabbit), Alexa Fluor 555 (Donkey Anti‐Mouse), Alexa Fluor 555 (Donkey Anti‐Rabbit), and Alexa Fluor 647 (Donkey Anti‐Chicken). Finally, the slides were covered with DAPI mounting medium (Invitrogen, Cat# P36935). Fluorescence signals were captured using a Carl Zeiss LSM 880 confocal microscope, as previously described [88].

### 4.17 Nitrite Assay

After HIV‐1 infection and cART treatment of hCOs, supernatants were collected, and nitrite levels were quantified using the Griess reagent kit (Cat# G7921) [89]. Briefly, 100 µL of supernatant was mixed with 100 µL of Griess reagent to generate a colorimetric reaction. After incubating for 30 min at room temperature, absorbance was measured at 540 nm. Nitrite concentrations were calculated using a sodium nitrite standard curve and expressed in µM.

### 4.18 RT‐PCR

Briefly, hCOs were homogenized, and RNA was extracted using Trizol reagent for semi‐quantitative RT‐PCR analysis. Total RNA was solubilized in RNase‐free water and quantified in duplicate by measuring the optical density (OD) at 260 nm. RNA purity was confirmed by examining the OD260/OD280 ratio using a Nanodrop Microvolume Spectrophotometer (Invitrogen). For cDNA synthesis, 2 µg of RNA was reverse transcribed using the iScript Advanced cDNA Synthesis Kit (Cat# 1725038, Bio‐Rad), following the manufacturer's instructions. RT‐qPCR was performed using diluted cDNA (1:20) and SYBR Green master mix. Samples were loaded into a 96‐well PCR plate and run on a CFX96 Real‐Time System (Bio‐Rad) with the following cycling conditions: initial denaturation at 95°C for 20–30 s (1 cycle), denaturation at 95°C for 1–3 s (40 cycles), annealing/extension at 60°C for 20 s (40 cycles), followed by a melt curve from 60 to 95°C with gradual temperature increase (1 cycle). Primers were purchased from BioRad. Gene expression levels were calculated by subtracting the target genes' cycle threshold (Ct) values from those of the housekeeping gene (GAPDH). Relative gene expression was calculated using the 2^−ΔΔCt^ method and reported as fold changes compared to the control group, as previously described [90].

### 4.19 Statistical Analysis

Statistical analyses were performed using GraphPad Prism version 8. Differences between the control, HIV‐infected, and cART‐treated groups (*n* = 3 independent experiments) were assessed using one‐way ANOVA followed by Tukey's multiple comparison test. Values are expressed as mean ± SD, with significance set at *p* < 0.05. Unless otherwise indicated, 6–8 individual organoids were pooled per experimental group for ELISA, RT‐qPCR, and Western blot analyses. All experiments were performed using organoids generated from at least three independent experiments. Gene set enrichment analysis was conducted using 10 000 permutations; pathways with a Benjamini‐Hochberg adjusted *p*‐value < 0.05 and a minimum of two genes were considered significantly enriched.

## Author Contributions

T.S. and K.C. designed and performed experiments, analyzed data, and drafted the manuscript. K.C. and M.S. performed Western blot experiments. J.C., S.G., and T.S. analyzed data and contributed to manuscript editing. A.A. and O.I. performed experiments and analyzed data. T.S., SNB., K.C., and J.C. provided critical scientific input. SNB. designed experiments and edited the manuscript. T.S. conceptualized, supervised, and supported the overall study, designed experiments, analyzed data, and co‐wrote the manuscript. All authors read and approved the final version of the manuscript.

## Funding

This work was supported by the National Institutes of Health (NIH)/National Institute on Drug Abuse (NIDA) under award R01 DA055568 and was partially supported by SEED Grants from Texas A&M University, College Station, Texas, USA. SNB was partially supported by National Institutes of Health (NIH)/National Institute on Drug Abuse (NIDA) grants DA059874 and DA061678.

## Conflicts of Interest

The authors declare no conflicts of interest.

## Supporting information




**Supporting File**: advs77954‐sup‐0001‐SuppMat.docx.

## Data Availability

The data that support the findings of this study are available on request from the corresponding author. The data are not publicly available due to privacy or ethical restrictions.

## References

[1] Global Task Team on HIV Targets Beyond 2025 , “Global HIV Targets: A Roadmap to 2030 and Beyond,” The Lancet (2025). 10.1016/S0140-6736(25)01449-7 40683288

[2] S. Badu Nyarko , J. A. Twum , A. S. Obeng , H. D. Djangba , O. Ryabinina , and F. Kyei , “Viral Kinetics Among Persons Living With HIV (PLWH) on Dolutegravir‐Based Antiretroviral Regimen: A Retrospective and Prospective Analysis from Selected HIV Clinics in Ghana,” PLoS ONE 20, no. 5 (2025): 0324360. 10.1371/journal.pone.0324360 PMC12091835 40392867

[3] D. Saylor , A. M. Dickens , N. Sacktor , et al., “HIV‐Associated Neurocognitive Disorder — Pathogenesis and Prospects for Treatment,” Nature Reviews Neurology 12, no. 4 (2016): 234–248. 10.1038/nrneurol.2016.27 PMC4937456 26965674

[4] R. S. Ziffra , C. N. Kim , J. M. Ross , et al., “Single‐Cell Epigenomics Reveals Mechanisms of Human Cortical Development,” Nature 598, no. 7879 (2021): 205–213. 10.1038/s41586-021-03209-8 PMC8494642 34616060

[5] Y. Sato , T. Asahi , and K. Kataoka , “Integrative Single‐Cell RNA‐Seq Analysis of Vascularized Cerebral Organoids,” BMC Biology 21, no. 1 (2023): 245. 10.1186/s12915-023-01711-1 PMC10634128 37940920

[6] M. A. Lancaster and J. A. Knoblich , “Generation of Cerebral Organoids From Human Pluripotent Stem Cells,” Nature Protocols 9, no. 10 (2014): 2329–2340. 10.1038/nprot.2014.158 PMC4160653 25188634

[7] R. S. Dos Reis , S. Sant , and V. Ayyavoo , “Three‐Dimensional Human Brain Organoids to Model HIV‐1 Neuropathogenesis,” Methods Molecular Biology 2610 (2023): 167–178. 10.1007/978-1-0716-2895-9_14 36534290

[8] W. Kong , J. Frouard , G. Xie et al., “Neuroinflammation Generated by HIV‐Infected Microglia Promotes Dysfunction and Death of Neurons in Human Brain Organoids,” PNAS Nexus 3, no. 5 (2024): pgae179. 10.1093/pnasnexus/pgae179 PMC11086946 38737767

[9] H. S. Kwon and S. H. Koh , “Neuroinflammation in Neurodegenerative Disorders: The Roles of Microglia And Astrocytes,” Translational Neurodegeneration 9, no. 1 (2020): 42. 10.1186/s40035-020-00221-2 PMC7689983 33239064

[10] S. R. Elizaldi , C. E. Hawes , A. Verma , et al., “Chronic SIV‐Induced Neuroinflammation Disrupts CCR^7+^ CD^4+^ T Cell Immunosurveillance in the Rhesus Macaque Brain,” Journal of Clinical Investigation 134, no. 9 (2024): 175332. 10.1172/JCI175332 PMC11060742 38470479

[11] C. A. Lee , A. J. Chavali , M. R. Harder , et al., “Simian Immunodeficiency Virus‐Infected Memory CD4(+) T Cells Infiltrate to the Site of Infected Macrophages in the Neuroparenchyma of a Chronic Macaque Model of Neurological Complications of AIDS,” mBio 11, no. 2 (2020): e03310–e03319. 10.1128/mBio.00602-20 PMC7175093 32317323

[12] E. K. Cartwright , D. Palesch , M. Mavigner , M. Paiardini , A. Chahroudi , and G. Silvestri , “Initiation of Antiretroviral Therapy Restores CD4+ T Memory Stem Cell Homeostasis in Simian Immunodeficiency Virus‐Infected Macaques,” Journal of Virology 90, no. 15 (2016): 6699–6708. 10.1128/JVI.00492-16 PMC4944279 27170752

[13] M. R. Simpson‐Abelson , G. Hernandez‐Mir , E. Childs , et al., “CCAAT/Enhancer‐Binding Protein β Promotes Pathogenesis of EAE,” Cytokine 92 (2017): 24–32. 10.1016/j.cyto.2017.01.005 PMC5337143 28088614

[14] C. Schwartz , J. Zhang , A. Leon‐Ponte , V. Nathan , R. J. Pomerantz , and T. M. Daniel , “Functional Interactions Between C/EBP, Sp1, and COUP‐TF Regulate Human Immunodeficiency Virus Type 1 Gene Transcription in Human Brain Cells,” Journal of Virology 74, no. 1 (2000): 65–73. 10.1128/jvi.74.1.65-73.2000 PMC111514 10590092

[15] S. Akira , H. Isshiki , T. Sugita , et al., “A Nuclear Factor for IL‐6 Expression (NF‐IL6) is a Member of a C/EBP Family,” The EMBO Journal 9, no. 6 (1990): 1897–1906. 10.1002/j.1460-2075.1990.tb08316.x PMC551896 2112087

[16] Y. Ren , W. Guo , and B. Qiao , “Abnormal Expression of CEBPB Promotes the Progression of Renal Cell Carcinoma through Regulating the Generation of IL‐6,” Heliyon 9, no. 10 (2023): 20175. 10.1016/j.heliyon.2023.e20175 PMC10520310 37767481

[17] S. Abraham , T. Sweet , B. E. Sawaya , J. Rappaport , K. Khalili , and S. Amini , “Cooperative Interaction of C/EBPβ and Tat Modulates MCP‐1 Gene Transcription in Astrocytes,” Journal of Neuroimmunology 160, no. 1‐2 (2005): 219–227. 10.1016/j.jneuroim.2004.11.009 15710476

[18] C.‐Y. Ko , W.‐L. Wang , S.‐M. Wang , Y.‐Y. Chu , W.‐C. Chang , and J.‐M. Wang , “Glycogen Synthase Kinase‐3β–Mediated CCAAT/Enhancer‐Binding Protein Delta Phosphorylation in Astrocytes Promot Es Migration and Activation of Microglia/Macrophages,” Neurobiology of Aging 35, no. 1 (2014): 24–34. 10.1016/j.neurobiolaging.2013.07.021 23993701

[19] C. Siebel and U. Lendahl , “Notch Signaling in Development, Tissue Homeostasis, and Disease,” Physiological Reviews 97, no. 4 (2017): 1235–1294. 10.1152/physrev.00005.2017 28794168

[20] E. Koutelou , S. Sato , C. Tomomori‐Sato , et al., “Neuralized‐Like 1 (Neurl1) Targeted to the Plasma Membrane by N‐Myristoylation Regulates the Notch Ligand Jagged1,” Journal of Biological Chemistry 283, no. 7 (2008): 3846–3853. 10.1074/jbc.M706974200 18077452

[21] S. Keerthivasan , R. Suleiman , R. Lawlor , et al., “Notch Signaling Regulates Mouse and Human Th17 Differentiation,” The Journal of Immunology 187, no. 2 (2011): 692–701. 10.4049/jimmunol.1003658 PMC3131467 21685328

[22] S. H. Patel , O. A. Ismaiel , W. R. Mylott , et al., “Cell‐Type Specific Differences in Antiretroviral Penetration and the Effects of HIV‐1 Tat and Morphine Among Primary Human Brain Endothelial Cells, Astrocytes, Pericytes, and Microglia,” Neuroscience Letters 712 (2019): 134475. 10.1016/j.neulet.2019.134475 PMC6941658 31491466

[23] L. Pellegrini , C. Bonfio , J. Chadwick , F. Begum , M. Skehel , and M. A. Lancaster , “Human CNS Barrier‐Forming Organoids With Cerebrospinal Fluid Production,” Science 369, no. 6500 (2020): aaz5626. 10.1126/science.aaz5626 PMC7116154 32527923

[24] S. L. Giandomenico , M. Sutcliffe , and M. A. Lancaster , “Generation and Long‐Term Culture of Advanced Cerebral Organoids for Studying Later Stages of Neural Development,” Nature Protocols 16, no. 2 (2021): 579–602. 10.1038/s41596-020-00433-w PMC7611064 33328611

[25] J. G. Walsh , S. N. Reinke , M. K. Mamik , et al., “Rapid Inflammasome Activation in Microglia Contributes to Brain Disease in HIV/AIDS,” Retrovirology 11, no. 1 (2014): 35. 10.1186/1742-4690-11-35 PMC4038111 24886384

[26] A. K. Min , B. Javidfar , R. Missall , et al., “HIV‐1 Infection of Genetically Engineered iPSC‐Derived central Nervous System‐Engrafted Microglia in a Humanized Mouse Model,” Journal of Virology 97, no. 12 (2023): 0159523. 10.1128/jvi.01595-23 PMC10734545 38032195

[27] X. Pan , H.‐M. Baldauf , O. T. Keppler , and O. T. Fackler , “Restrictions to HIV‐1 Replication in Resting CD^4+^ T Lymphocytes,” Cell Research 23, no. 7 (2013): 876–885. 10.1038/cr.2013.74 PMC3698640 23732522

[28] V. R. Rao , U. Neogi , J. S. Talboom , et al., “Clade C HIV‐1 Isolates Circulating in Southern Africa Exhibit a Greater Frequency of Dicysteine Motif‐Containing Tat Variants Than Those in Southeast Asia and Cause Increased Neurovirulence,” Retrovirology 10, no. 1 (2013): 61. 10.1186/1742-4690-10-61 PMC3686704 23758766

[29] H. Schmidtmayerova , M. Alfano , G. Nuovo , and M. Bukrinsky , “Human Immunodeficiency Virus Type 1 T‐Lymphotropic Strains Enter Macrophages via a CD4‐ and CXCR4‐mediated Pathway: Replication Is Restricted at a Postentry Level,” Journal of Virology 72, no. 6 (1998): 4633–4642. 10.1128/jvi.72.6.4633-4642.1998 PMC109980 9573226

[30] K. Zhang , J. D. Hocker , M. Miller , et al., “A Single‐Cell Atlas of Chromatin Accessibility in the Human Genome,” Cell 184, no. 24 (2021): 5985–6001.e19. 10.1016/j.cell.2021.10.024 PMC8664161 34774128

[31] Y. Ma , S. Sun , X. Shang , E. T. Keller , M. Chen , and X. Zhou , “Integrative Differential Expression and Gene Set Enrichment Analysis Using Summary Statistics for scRNA‐seq Studies,” Nature Communications 11, no. 1 (2020): 1585. 10.1038/s41467-020-15298-6 PMC7101316 32221292

[32] H. S. Fox , M. Niu , B. M. Morsey , et al., “Morphine Suppresses Peripheral Responses and Transforms Brain Myeloid Gene Expression to Favor Neuropathogenesis in SIV Infection,” Frontiers in Immunology 13 (2022): 1012884. 10.3389/fimmu.2022.1012884 PMC9709286 36466814

[33] R. M. Oguariri , T. W. Brann , J. W. Adelsberger , et al., “Short Communication: S100A8 and S100A9, Biomarkers of SARS‐CoV‐2 Infection and Other Diseases, Suppress HIV Replication in Primary Macrophages,” AIDS Research and Human Retroviruses 38, no. 5 (2022): 401–405. 10.1089/aid.2021.0193 PMC9131038 35045753

[34] G. Maarifi , J. Lagisquet , Q. Hertel , et al., “Alarmin S100A9 Restricts Retroviral Infection by Limiting Reverse Transcription in human Dendritic Cells,” The EMBO Journal 40, no. 16 (2021): 106540. 10.15252/embj.2020106540 PMC8365256 34121210

[35] R. Sartorius , M. Trovato , R. Manco , L. D'Apice , and P. De Berardinis , “Exploiting Viral Sensing Mediated by Toll‐like Receptors to Design Innovative Vaccines,” NPJ Vaccines 6, no. 1 (2021): 127. 10.1038/s41541-021-00391-8 PMC8553822 34711839

[36] H.‐S. Suh , M.‐L. Zhao , N. Choi , T. J. Belbin , C. F. Brosnan , and S. C. Lee , “TLR3 and TLR4 Are Innate Antiviral Immune Receptors in Human Microglia: Role of IRF3 in Modulating Antiviral and Inflammatory Response in the CNS,” Virology 392, no. 2 (2009): 246–259. 10.1016/j.virol.2009.07.001 PMC2752833 19646728

[37] N. Sacktor , R. L. Skolasky , E. Seaberg , et al., “Prevalence of HIV‐Associated Neurocognitive Disorders in the Multicenter AIDS Cohort Study,” Neurology 86, no. 4 (2016): 334–340. 10.1212/WNL.0000000000002277 PMC4776086 26718568

[38] D. B. Clifford and B. M. Ances , “HIV‐Associated Neurocognitive Disorder,” The Lancet Infectious Diseases 13, no. 11 (2013): 976–986. 10.1016/S1473-3099(13)70306-2 24156889

[39] L. Huangfu , R. Li , Y. Huang , and S. Wang , “The IL‐17 Family in Diseases: From Bench to Bedside,” Signal Transduction and Targeted Therapy 8, no. 1 (2023): 402. 10.1038/s41392-023-01620-3 PMC10564932 37816755

[40] L. Sun , J. Wu , F. Du , X. Chen , and Z. J. Chen , “Cyclic GMP‐AMP Synthase Is a Cytosolic DNA Sensor That Activates the Type I Interferon Pathway,” Science 339, no. 6121 (2013): 786–791. 10.1126/science.1232458 PMC3863629 23258413

[41] L. L. P. de Lima , A. Q. T. de Oliveira , T. C. F. Moura , et al., “STING and cGAS Gene Expressions Were Downregulated Among HIV‐1‐Infected Persons after Antiretroviral Therapy,” Virology journal 18, no. 1 (2021): 78. 10.1186/s12985-021-01548-6 PMC8047565 33858455

[42] J. Rasaiyaah , C. P. Tan , A. J. Fletcher , et al., “HIV‐1 Evades Innate Immune Recognition Through Specific Cofactor Recruitment,” Nature 503, no. 7476 (2013): 402–405. 10.1038/nature12769 PMC3928559 24196705

[43] M. Doke , J. P. McLaughlin , J. J. Cai , et al., “HIV‐1 Tat and Cocaine Impact Astrocytic Energy Reservoirs and Epigenetic Regulation by Influencing the LINC01133‐hsa‐miR‐4726‐5p‐NDUFA9 Axis,” Molecular Therapy ‐ Nucleic Acids 29 (2022): 243–258. 10.1016/j.omtn.2022.07.001 PMC9307901 35892093

[44] Y. Hao , X. Yang , D. Zhang , J. Luo , and R. Chen , “Long Noncoding RNA LINC01186, Regulated by TGF‐β/SMAD3, Inhibits Migration and Invasion Through Epithelial‐Mesenchymal‐Transition in Lung Cancer,” Gene 608 (2017): 1–12. 10.1016/j.gene.2017.01.023 28119085

[45] M. Y. Zheng and L. Z. Luo , “The Role of IL‐17A in Mediating Inflammatory Responses and Progression of Neurodegenerative Diseases,” International Journal of Molecular Sciences 26, no. 6 (2025): 2505. 10.3390/ijms26062505 PMC11941770 40141149

[46] C. Guo , L. Sun , X. Chen , and D. Zhang , “Oxidative Stress, Mitochondrial Damage and Neurodegenerative Diseases,” Neural Regeneration Research 8, no. 21 (2013): 2003–2014. 10.3969/j.issn.1673-5374.2013.21.009 PMC4145906 25206509

[47] J. Chapuis , F. Hansmannel , M. Gistelinck , et al., “Increased Expression of BIN1 Mediates Alzheimer Genetic Risk by Modulating Tau Pathology,” Molecular Psychiatry 18, no. 11 (2013): 1225–1234. 10.1038/mp.2013.1 PMC3807661 23399914

[48] I. C. Anthony , S. N. Ramage , F. W. Carnie , P. Simmonds , and J. E. Bell , “Accelerated Tau Deposition in the Brains of Individuals Infected With Human Immunodeficiency Virus‐1 Before and After the Advent of Highly Active Anti‐Retroviral Therapy,” Acta Neuropathologica 111, no. 6 (2006): 529–538. 10.1007/s00401-006-0037-0 16718349

[49] E. A. Eugenin , J. E. Clements , M. C. Zink , and J. W. Berman , “Human Immunodeficiency Virus Infection of Human Astrocytes Disrupts Blood‐Brain Barrier Integrity by a Gap Junction‐Dependent Mechanism,” Journal of Neuroscience 31, no. 26 (2011): 9456–9465. 10.1523/JNEUROSCI.1460-11.2011 PMC3132881 21715610

[50] B. N. Phan , M. H. Ray , X. Xue , et al., “Single Nuclei Transcriptomics in Human and Non‐Human Primate Striatum in Opioid Use Disorder,” Nature Communications 15, no. 1 (2024): 878. 10.1038/s41467-024-45165-7 PMC10831093 38296993

[51] X. Xu , M. Niu , B. G. Lamberty , K. Emanuel , L. D. Estrella , and H. S. Fox , “T Cell‐Mediated SIV Dissemination into the CNS: A Single‐Cell Transcriptomic Analysis,” Journal of Neuroinflammation 22, no. 1 (2025): 226. 10.1186/s12974-025-03543-y PMC12509402 41063314

[52] M. K. Ash , L. Al‐Harthi , and J. R. Schneider , “HIV in the Brain: Identifying Viral Reservoirs and Addressing the Challenges of an HIV Cure,” Vaccines (Basel) 9 (2021): 867. 10.3390/vaccines9080867 PMC8402376 34451992

[53] J. B. Honeycutt , B. Liao , C. C. Nixon , et al., “T Cells Establish and Maintain CNS Viral Infection in HIV‐Infected Humanized Mice,” Journal of Clinical Investigation 128, no. 7 (2018): 2862–2876. 10.1172/JCI98968 PMC6026008 29863499

[54] S. Selvam , R. S. Dos Reis , M. C. E. Wagner , R. Krishnakumar , K. Keshavarz , M. R. Ebrahimkhani , and V. Ayyavoo , “Vascularized Human Brain Organoids as a Model of the Brain‐Peripheral axis in HIV‐1 neuropathogenesis,” Scientific Reports 16, no. 1 (2026): 3794, 10.1038/s41598-025-33940-5. PMC12852793 41491209

[55] Y. Sui , R. Potula , N. Dhillon , et al., “Neuronal Apoptosis Is Mediated by CXCL10 Overexpression in Simian Human Immunodeficiency Virus Encephalitis,” The American Journal of Pathology 164, no. 5 (2004): 1557–1566. 10.1016/S0002-9440(10)63714-5 PMC1615658 15111302

[56] D. M. P. Lawrence , P. Seth , L. Durham , et al., “Astrocyte Differentiation Selectively Upregulates CCL2/Monocyte Chemoattractant Protein‐1 in Cultured Human Brain‐Derived Progenitor Cells,” Glia 53, no. 1 (2006): 81–91. 10.1002/glia.20261 16206198

[57] D. P. Ramji and P. Foka , “CCAAT/Enhancer‐Binding Proteins: Structure, Function and Regulation,” Biochemical Journal 365, no. 3 (2002): 561–575. 10.1042/BJ20020508 PMC1222736 12006103

[58] A. Maitra , F. Shen , W. Hanel , et al., “Distinct Functional Motifs Within the IL‐17 Receptor Regulate Signal Transduction and Target Gene Expression,” Proceedings of the National Academy of Sciences 104, no. 18 (2007): 7506–7511. 10.1073/pnas.0611589104 PMC1863505 17456598

[59] R. T. Gandhi , R. J. Landovitz , P. E. Sax , et al., “Antiretroviral Drugs for Treatment and Prevention of HIV in Adults: 2024 Recommendations of the International Antiviral Society–USA Panel,” Jama 333, no. 7 (2025): 609–628. 10.1001/jama.2024.24543 39616604

[60] P. Filippidis and S. F. Farhadian , “Smoldering in the Sanctuary: HIV‐Associated Brain Injury in the ART Era,” Journal of Clinical Investigation 136, no. 9 (2026): 204831. 10.1172/JCI204831 PMC13132375 42065243

[61] S. Martinez‐Meza , T. A. Premeaux , S. M. Cirigliano , et al., “Antiretroviral Drug Therapy Does Not Reduce Neuroinflammation in an HIV‐1 Infection Brain Organoid Model,” Journal of Neuroinflammation 22, no. 1 (2025): 66. 10.1186/s12974-025-03375-w PMC11881274 40045391

[62] T. M. Temu , J. S. Zifodya , S. J. Polyak , et al., “Antiretroviral Therapy Reduces but Does Not Normalize Immune and Vascular Inflammatory Markers in Adults With Chronic HIV Infection in Kenya,” Aids 35, no. 1 (2021): 45–51. 10.1097/QAD.0000000000002729 PMC7718419 33055570

[63] C. O. Hileman and N. T. Funderburg , “Inflammation, Immune Activation, and Antiretroviral Therapy in HIV,” Current HIV/AIDS Reports 14, no. 3 (2017): 93–100. 10.1007/s11904-017-0356-x PMC5514315 28434169

[64] A. Borjabad , S. Morgello , W. Chao , et al., “Significant Effects of Antiretroviral Therapy on Global Gene Expression in Brain Tissues of Patients With HIV‐1‐Associated Neurocognitive Disorders,” PLoS Pathogens 7, no. 9 (2011): 1002213. 10.1371/journal.ppat.1002213 PMC3164642 21909266

[65] H. D. Nguyen and W. K. Kim , “New Genetic Insights into HIV‐Associated Neurocognitive Disorder and Alzheimer's Disease,” Genes & Diseases 12, no. 5 (2025): 101576. 10.1016/j.gendis.2025.101576 PMC12142519 40485978

[66] S. Canchi , M. K. Swinton , R. A. Rissman , and J. A. Fields , “Transcriptomic Analysis of Brain Tissues Identifies a Role for CCAAT Enhancer Binding Protein β in HIV‐Associated Neurocognitive Disorder,” Journal of Neuroinflammation 17, no. 1 (2020): 112. 10.1186/s12974-020-01781-w PMC7149918 32276639

[67] J. Nitkiewicz , A. Borjabad , S. Morgello , et al., “HIV Induces Expression of Complement Component C3 in Astrocytes by NF‐κB‐Dependent Activation of Interleukin‐6 Synthesis,” Journal of Neuroinflammation 14, no. 1 (2017): 23. 10.1186/s12974-017-0794-9 PMC5267445 28122624

[68] Z. Liu , A.‐W. Qiu , Y. Huang , et al., “IL‐17A Exacerbates Neuroinflammation and Neurodegeneration By Activating Microglia in Rodent Models of Parkinson's Disease,” Brain, Behavior, and Immunity 81 (2019): 630–645. 10.1016/j.bbi.2019.07.026 31351185

[69] D. L. Irons , T. Meinhardt , C. Allers , M. J. Kuroda , and W.‐K. Kim , “Overexpression and Activation of Colony‐Stimulating Factor 1 Receptor in the SIV/Macaque Model of HIV Infection and neuroHIV,” Brain Pathology 29, no. 6 (2019): 826–836. 10.1111/bpa.12731 PMC6810716 31033097

[70] L. Bo and X. Bo , “Colony Stimulating Factor 1: Friend or Foe of Neurons?,” Neural Regeneration Research 17, no. 4 (2022): 773–774. 10.4103/1673-5374.322451 PMC8530122 34472465

[71] S. Guo , H. Wang , and Y. Yin , “Microglia Polarization From M1 to M2 in Neurodegenerative Diseases,” Frontiers in Aging Neuroscience 14 (2022): 815347. 10.3389/fnagi.2022.815347 PMC8888930 35250543

[72] K. Taal , J. Tuvikene , G. Rullinkov , et al., “Neuralized Family Member NEURL1 Is a Ubiquitin Ligase for the cGMP‐Specific Phosphodiesterase 9A,” Scientific Reports 9, no. 1 (2019): 7104. 10.1038/s41598-019-43069-x PMC6506465 31068605

[73] N. Teider , D. K. Scott , A. Neiss , et al., “Neuralized1 Causes Apoptosis and Downregulates Notch Target Genes in Medulloblastoma,” Neuro‐Oncology 12, no. 12 (2010): 1244–1256. 10.1093/neuonc/noq091 PMC3018940 20847082

[74] W. Wang , F. Zhao , X. Ma , G. Perry , and X. Zhu , “Mitochondria Dysfunction in the Pathogenesis of Alzheimer's Disease: Recent Advances,” Molecular Neurodegeneration 15, no. 1 (2020): 30. 10.1186/s13024-020-00376-6 PMC7257174 32471464

[75] M. T. Henrich , W. H. Oertel , D. J. Surmeier , and F. F. Geibl , “Mitochondrial Dysfunction in Parkinson's Disease – A Key Disease Hallmark With Therapeutic Potential,” Molecular Neurodegeneration 18, no. 1 (2023): 83. 10.1186/s13024-023-00676-7 PMC10640762 37951933

[76] J. Zhao , M. Schank , L. Wang , et al., “Mitochondrial Functions Are Compromised in CD4 T Cells from ART‐Controlled PLHIV,” Frontiers in Immunology 12 (2021): 658420. 10.3389/fimmu.2021.658420 PMC8129510 34017335

[77] Y. Xia , Z.‐H. Wang , J. Zhang , et al., “C/EBPβ Is a Key Transcription Factor for APOE and Preferentially Mediates ApoE4 Expression in Alzheimer's Disease,” Molecular Psychiatry 26, no. 10 (2021): 6002–6022. 10.1038/s41380-020-00956-4 PMC8758498 33339957

[78] L. A. Brown , J. Scarola , A. J. Smith , P. R. Sanberg , J. Tan , and B. Giunta , “The Role of Tau Protein in HIV‐Associated Neurocognitive Disorders,” Molecular Neurodegeneration 9, no. 1 (2014): 40. 10.1186/1750-1326-9-40 PMC4210623 25304757

[79] O. Saha , A. R. Melo de Farias , A. Pelletier , et al., “The Alzheimer's Disease Risk Gene BIN1 Regulates Activity‐Dependent Gene Expression in Human‐Induced Glutamatergic Neurons,” Molecular Psychiatry 29, no. 9 (2024): 2634–2646. 10.1038/s41380-024-02502-y PMC11420064 38514804

[80] A. Altmann , L. M. Aksman , N. P. Oxtoby , et al., “Towards Cascading Genetic Risk in Alzheimer's Disease,” Brain 147, no. 8 (2024): 2680–2690. 10.1093/brain/awae176 PMC11292901 38820112

[81] L. Vallés‐Saiz , J. Ávila , F. Hernández , and Lamivudine , “(3TC), a Nucleoside Reverse Transcriptase Inhibitor, Prevents the Neuropathological Alterations Present in Mutant Tau Transgenic Mice,” International Journal of Molecular Sciences 24, no. 13 (2023): 11144. 10.3390/ijms241311144 PMC10342792 37446327

[82] P. K. Dash , S. Gorantla , H. E. Gendelman , et al., “Loss of Neuronal Integrity During Progressive HIV‐1 Infection of Humanized Mice,” The Journal of Neuroscience 31, no. 9 (2011): 3148–3157. 10.1523/JNEUROSCI.5473-10.2011 PMC3059783 21368026

[83] J. S. Fleck , S. M. J. Jansen , D. Wollny , et al., “Inferring and Perturbing Cell Fate Regulomes in Human Brain Organoids,” Nature 621, no. 7978 (2023): 365–372. 10.1038/s41586-022-05279-8 PMC10499607 36198796

[84] G. E. Hoffman , et al., “Efficient Differential Expression Analysis of Large‐Scale Single Cell Transcriptomics Data Using Dreamlet,” bioRxiv, 2024: p.2023.03.17.533005. 10.1038/s41467-026-75680-8 PMC13601538 42778542

[85] C. Hu , T. Li , Y. Xu , et al., “CellMarker 2.0: An Updated Database of Manually Curated Cell Markers in human/Mouse and Web Tools Based on scRNA‐seq Data,” Nucleic Acids Research 51, no. D1 (2023): D870–D876. 10.1093/nar/gkac947 PMC9825416 36300619

[86] I. Ahmad , S. Gupta , P. Faulkner , et al., “Single‐Nucleus Transcriptomics of Epicardial Adipose Tissue from Female Pigs Reveals Effects of Exercise Training on Resident Innate and Adaptive Immune Cells,” Cell Communication and Signaling 22, no. 1 (2024): 243. 10.1186/s12964-024-01587-w PMC11046969 38671495

[87] I. Jamal , A. Paudel , L. Thompson , M. Abdelmalek , I. A. Khan , and V. B. Singh , “Sulforaphane Prevents the Reactivation of HIV‐1 by Suppressing NFκB Signaling,” Journal of Virus Eradication 9, no. 3 (2023): 100341. 10.1016/j.jve.2023.100341 PMC10469555 37663574

[88] J. Haorah , H. Iyappan , M. Samikkannu , K. Chennakesavan , J. P. McLaughlin , and T. Samikkannu , “Epigenetics and Mitochondrial Biogenesis: The Role of Sirtuins in HIV Neuropathogenesis,” Molecular Neurobiology 62, no. 8 (2025): 10333–10348. 10.1007/s12035-025-04885-7 PMC12289736 40198445

[89] L. A. Ridnour , J. E. Sim , M. A. Hayward , et al., “A Spectrophotometric Method for the Direct Detection and Quantitation of Nitric Oxide, Nitrite, and Nitrate in Cell Culture Media,” Analytical Biochemistry 281, no. 2 (2000): 223–229. 10.1006/abio.2000.4583 10870839

[90] C. Karthick , S. Nithiyanandan , M. M. Essa , G. J. Guillemin , S. K. Jayachandran , and M. Anusuyadevi , “Time‐Dependent Effect of Oligomeric Amyloid‐β (1–42)‐Induced Hippocampal Neurodegeneration in Rat Model of Alzheimer's Disease,” Neurological Research 41, no. 2 (2019): 139–150. 10.1080/01616412.2018.1544745 30453864

