# Supplementary material for: Molecular Signatures of HIV‐Induced Neuroinflammation and Neurodegeneration in Human Cerebral Organoids: Identification of Therapeutic Targets for NeuroHIV
Source: Adv Sci (Weinh). 2026 Oct 18:e77954. Online ahead of print. doi: 10.1002/advs.77954 (PMC13676815; doi:10.1002/advs.77954)
Supplement: Supplementary file 1 [file ADVS-9999-e77954-s001.docx]

Molecular Signatures of HIV-Induced Neuroinflammation and Neurodegeneration in Human Cerebral Organoids: Identification of Therapeutic Targets for NeuroHIV

**Karthick Chennakesavan^1^, Malaroviyam Samikkannu^1^, Shreyan Gupta^2^, Olasunkanmi Israel Tayo^3^, Arpan Acharya^3^, James J. Cai^2^, Siddappa N. Byrareddy^3^, and Thangavel Samikkannu^1^***

^1^Department of Pharmaceutical Sciences, Irma Lerma Rangel College of Pharmacy, Texas A&M University Health Science Center, College Station, Texas -77843, USA.

^2^Department of Veterinary Integrative Biosciences, School of Veterinary Medicine and Biomedical Sciences, Texas A&M University, College Station, Texas -77843, USA.

^3^Department of Pharmacology and Experimental Neuroscience, College of Medicine, University of Nebraska Medical Center, Omaha, NE, USA

***Corresponding Author:**

Thangavel Samikkannu, Ph.D.

Associate Professor

Department of Pharmaceutical Sciences

Irma Lerma Rangel College of Pharmacy

Texas A&M University

College Station

Texas-77845, USA.

Tel: 979-436-9916

Fax: 979-436-0087

Email: [thangavel@tamu.edu](mailto:thangavel@tamu.edu)

**Supplementary table S1: List of Antibodies used for Western blot analysis**

| **Antibody** | **Company** | **Cat #** |
| --- | --- | --- |
| TLR 4 | Protein Tech | 66350-1-Ig |
| C3/C3b/C3c | Protein Tech | 21337-1-AP |
| CD 24 | Protein Tech | 67627-1-Ig |
| IL-17A | Protein Tech | 66148-1-Ig |
| IFN - gamma | Protein Tech | 15365-I-AP |
| TGF-β (56E4) | Cell Signaling | 3709T |
| IL-1 β | Protein Tech | 16806-1-AP |
| Cleaved caspase-1 (Asp297) (D57A2) | Cell Signaling | 4199T |
| Cleaved-IL-1β (Asp116) (D3A3Z) | Cell Signaling | 83186T |
| iNOS | Thermo Fisher Scientific | MA5-17139 |
| cGAS | Santa Cruz Biotechnology | SC-515777 |
| TMEM173/ STING | Protein Tech | 19851-1-AP |
| Stat1 (D1K9Y) | Cell Signaling | 14994T |
| Phospho-Stat1 (Tyr701) (58D6) | Cell Signaling | 9167T |
| PGC1a | Protein Tech | 66369-I-IG |
| NDUFB9 (E7W6K) | Cell Signaling | 99235T |
| NDUFA9 | ABclonal | A3196 |
| FKBP8 | Protein Tech | 11173-1-AP |
| AMBRA1 | Protein Tech | 13762-1-AP |
| Phospho-AMPK alpha1/2 (Thr183/172) | Elabscience | AB-21121 |
| AMPK alpha | Protein Tech | 10929-2-AP |
| OXPHOS | Abcam | ab110411 |
| TAU | ABclonal | A23490 |
| Phospho-Tau (Ser202, Thr205) | Thermo Fisher Scientific | MN1020 |
| MAP2 | Thermo Fisher Scientific | PA1-10005 |
| NF-κB p65 | Protein Tech | 10745-1-AP |
| Anti-CSF1R antibody [EPR21885-161] | Abcam | ab221684 |
| PSD-95 | Thermo Fisher Scientific | 51-6900 |
| Anti-Iba1/AIF1 | Millipore Sigma | MABN92 |
| Beta Actin | Cell Signaling | 12262S |

**Supplementary table S2: List of Antibodies used for immunocytochemistry**

| **Antibody** | **Company** | **Cat #** |
| --- | --- | --- |
| CD68 | Cell Signaling | 76437T |
| Anti-Iba1/AIF1 | Millipore Sigma | MABN92 |
| FOXO3A | Protein Tech | 10849-1-AP |
| TAU | ABclonal | A23490 |
| Phospho-Tau (Ser202, Thr205) | Thermo Fisher Scientific | MN1020 |
| SOX2 | Thermo Fisher Scientific | A11501 |
| MAP2 | Thermo Fisher Scientific | PA1-10005 |
| PAX 6 | ABclonal | A7334 |
| Nestin | ABclonal | A11861 |
| BIN1 (E4A1P) | Cell Signaling | 51844S |
| Ki67 | ABclonal | A2094 |
| GFAP | ABclonal | A0237 |
| FOXG1 | ABclonal | A16851 |
| TNF alpha | Thermo Fisher Scientific | PA1-40281 |
| Anti-Beta-Tubulin III Antibody | Stemcell Technologies | 60052 |
| PSD-95 | Thermo Fisher Scientific | 51-6900 |
| HIV-1 Gag p24 | Fisher scientific | MAB73601SP |
|  |  |  |

**Figure Legends**

**Supplementary Fig. S1:** Immunostaining revealed dynamic temporal expression of neural progenitor and forebrain-specific markers in developing hCOs. (A) MAP2, a marker of mature neurons (B) FoxG1, a transcription factor critical for forebrain identity, was analyzed at different developmental stages (day 15, 30, and 60). Scale bar: 50μm.

**Supplementary Fig. S2**: **Transcriptional Profile of hCOs and HIV-1 infected hCOs.** Functional enrichment analysis of differentially expressed genes. (**A)** Top 10 GO terms for the DEGs based on snRNA seq (BPs). (**B)** Top 10 GO terms for DEGS based on ATAC scores from different cell type populations of HIV-1 vs Control. GO, Gene Ontology; BP, biological process; adjusted p-value.

**Supplementary Fig. S3:** (A) Enrichr analysis was performed to identify the most significantly enriched Gene Ontology (GO) biological process terms. The enriched GO terms in HIV-1-infected human cerebral organoids (hCOs) revealed interactions among the IL-17A, CEBPB, STAT1, and CSF1R signaling pathways. (B) Visualization of the top enriched GO biological process pathways associated with upregulated differentially expressed genes (DEGs) across the major cell types in HIV-1-infected hCOs.

**Supplementary Fig. S4:** (A-H) Volcano plot of significant genes (P ≤ 0.05; Wilcoxon rank sum test, Bonferroni-adjusted) different when comparing HIV-1 infected vs. Control (A-D) and HIV-1 + cART vs. HIV-1 infection (E-H) from neurons and oligodendrocytes.

**Supplementary Fig. S5:** Single-nucleus ATAC-seq profiling of cerebral organoids across control, HIV-1–infected, and cART-treated conditions identifies condition-specific changes in chromatin accessibility, with increased peak intensities indicating enhanced regulatory activity. Analysis was performed across major cell types, including neurons and oligodendrocytes. Differential peak analysis revealed dynamic changes in regulatory elements, particularly at loci associated with immune-related transcription factors, including NFKB2 (targeted gene locus on Chr 10), NEURL1 locus on Chr 10, CEBPB locus on Chr 20, and STAT1 locus on Chr 2.

**Supplementary Fig. S6:** Representative sequencing tracks for the IFI6, IRF3, IRF8, TFEB, STAT3, STAT4, S100A8, and S100A9 gene loci show distinct ATAC-seq peaks at the promoter and the known enhancer in hCOs. The ATAC-seq data have been normalized to take sequencing depth into account, and the scale on the y-axis was chosen for optimal visualization of peaks for each cell type.

**Supplementary Fig. S7: (**A) Functional enrichment network showing key genes and associated biological processes and phenotypes. Green nodes indicate key genes, purple/pink nodes represent enriched pathways and GO terms, and orange nodes indicate phenotype-associated terms. Edges represent functional associations between genes and enriched terms. (B) Cell-type-specific functional enrichment networks in oligodendrocytes, cRGCs, neurons, and neural stem cells (NSCs). Networks highlight cell-type-specific genes and enriched pathways related to immune response, inflammatory signaling, T-cell activation, receptor signaling, neurogenesis, and canonical Wnt signaling. Node size represents gene number, and color indicates fold change.

**Supplementary Fig. S8:** Immunostaining of microglia/macrophages activation marker CD68 in HIV-1-infected hCOs compared to control and cART treatment. Scale bar: 50μm.

**Supplementary Fig. S9:** Representative immunofluorescence images showing the co-localization of microglia and IL-17A in control, HIV-1-infected, and cART-treated human cerebral organoids (hCOs). HIV-1-infected hCOs exhibited increased co-localization of microglia and IL-17A expression compared with control and cART-treated groups. Microglia were identified using P2RY12 immunostaining (red), IL-17A was detected using anti-IL-17A antibodies (green), and nuclei were counterstained with DAPI (blue). Scale bar = 50 μm.

**Supplementary Fig. S10:** GO enrichment analysis of the astrocytes, neurons, mDN, NSCs, Oligodendrocytes, and cRGC linc-RNA nearby genes. Nearby genes were collected within 100 kb up- or downstream of lincRNAs related to TGF-β signaling and oxidative phosphorylation between each group.

**Supplementary Fig. S11.** Representative immunofluorescence images showing NEURL1 and β-tubulin III (Tuj1) expression in control, HIV-1-infected, and cART-treated human cerebral organoids (hCOs). NEURL1 was detected using anti-NEURL1 antibodies (green), whereas β-tubulin III (Tuj1), a neuronal marker, was detected using anti-β-tubulin III antibodies (red). Nuclei were counterstained with DAPI (blue). Scale bar = 50 μm.

**Supplementary Fig. S12:** HIV-infected brain organoids induce Tau pathology and its potential link to HAND. (**A)** Enriched KEGG pathway. (**B)** KEGG enrichment of the Top signal pathway in HIV infected hCOs within the microglia populations.

**Supplementary Fig. S13:** Functional enrichment analysis of biological processes in the macaque brain. (**A-D)** Cell type-specific functional enrichment analysis of biological processes using upregulated immune-related genes in the macaque brain. GO Biological Process pathways were analyzed using fgsea with ranked gene lists (log_2_FC ≥ 1, p < 0.05, ≥5% expression). Significant pathways (nominal p < 0.05) were visualized as dot plots. (**E-H).** Selected genes of specific pathways, including chemokine signaling, TGF-β signaling, type I interferon signaling, and oxidative phosphorylation, were presented as dot plots, where the diameter of the dots indicates the percentage of cells in which the specific genes were expressed. NES: Normalized enrichment scores.

**Supplementary Fig. S14:** Gene Set Enrichment Analysis (GSEA) of the top enriched pathways in astrocytes (**A),** oligodendrocytes **(B),** neurons (**C),** and microglia (**D)** from control and SIV+ART-treated RMs. The size of the dots indicates the number of genes involved in each pathway. NES: Normalized enrichment scores.

**Full blot images**

Full Blot images: To conserve samples and maintain experimental consistency, certain membranes were reprobed, and the same β-actin blot was reused as the loading control.
